# Assessing knowledge, attitudes, and practices toward sexually transmitted infections among Baghdad undergraduate students for research-guided sexual health education

**DOI:** 10.3389/fpubh.2023.1017300

**Published:** 2023-02-16

**Authors:** Ghaith Al-Gburi, Ali Al-Shakarchi, Jaafar D. Al-Dabagh, Faris Lami

**Affiliations:** ^1^College of Medicine, University of Baghdad, Baghdad, Iraq; ^2^Department of Community and Family Medicine, College of Medicine, University of Baghdad, Baghdad, Iraq

**Keywords:** sexually transmitted infections, sex education, Middle East, Iraq, Baghdad

## Abstract

**Background:**

Sexually transmitted infections are common and tend to cause a lot of public misconceptions. This study was conducted to identify knowledge gaps and negative attitudes toward sexually transmitted infections and infected individuals among undergraduate students and give recommendations accordingly for the development of more objective research-guided health campaigns and school sex education programs.

**Method:**

A cross-sectional study was conducted between May 17, 2022 and June 2, 2022 using a self-administrated questionnaire containing 84 items related to sexually transmitted infections distributed online to Baghdad-based university students.

**Result:**

The sample consisted of 823 respondents; 332 men and 491 women. Overall knowledge was moderate to high, with 628 individuals (76.3%) answering more than half the questions correctly. There was no difference according to gender or previous sexual experience, but knowledge increased by an average of 2.73 points (*p* < 0.001) when a participant knew a previously infected individual. Less than half identified systemic symptoms of STIs, and their knowledge of other HIV items was also poor. Most respondents (85.5%) agreed to the need for sex education during middle or high school and cited traditional barriers as the most critical barrier (64.8%); in comparison, those who did not agree on its need cited the sensitivity of the subject (40.3%) or religious barriers (20.2%) as more important.

**Conclusion:**

Specific knowledge gaps exist for HIV and non-HIV sexually transmitted infections; these should be addressed during sex education, focusing on specific high-risk groups. Negative attitudes and stigmatizing behavior should be addressed as well by increasing focused STI knowledge.

## Introduction

Sexually transmitted infections (STIs) are a major public health concern worldwide. They refer to the multiplication of microbes in the genital tract after transmission through sexual routes. They can present with a broad spectrum of manifestations ranging from asymptomatic infections to diseases, which can end with infertility, cancers, and even death. According to the WHO, more than one million STIs are acquired every day globally (1). In developed countries, like the USA, nearly 27 million new STIs are estimated to be transmitted annually (2), half of which are among youth aged 15–24 years. While in the UK, the highest rate of more than 142,000 diagnosed cases was recorded among the same age group in 2020 (3). The estimated incidence for four curable STIs (chlamydia, gonorrhea, syphilis, and trichomoniasis) was 60.6 per 1,000 in the Middle East/North Africa region. Although this region is classified as low risk, the estimated death of 10.4 per million was more than regions with higher incidence (4). STIs Epidemiological data (in terms of incidence and prevalence) from Iraq was scarce and incomplete; with more focus on AIDS (5) or using a syndromic approach for estimation rather than laboratory tests (6).

Despite the incomplete profile of STIs in Iraq, the recent political, socioeconomic, and cultural situation of the country suggests that there are factors that may contribute to an increased spread of STIs and cause under-reporting. These factors may include war, displacement, poverty, unemployment, and the disruption of families and communities. Furthermore, increased access to the outside world through TV, cell phones, and the Internet may lead to the development of a more open mindset toward sex in a large proportion of the young Iraqi population (5), especially in urban regions including the capital city of Baghdad. University students might be especially vulnerable, as they are part of the youth group (15–24 years) designated by the United Nations; these individuals are usually disproportionately high in terms of their STI incidence (3). In Iraq, university also characterizes a period of return to mixed-gender education after 6 years of segregation during middle and high school. A sharp increase in high-risk sexual behavior is therefore expected.

According to Becker's health belief model, people's knowledge and attitude toward a health-related problem might correlate with their future behavior (7); this, in turn, means that studies which assess these indices can be useful during the development and implementation of public health campaigns and education programs in a way that is suitable to the needs of the local environment. Many studies of this nature have been performed in countries that are culturally similar to Iraq; like Iran (8, 9), Saudi Arabia (10, 11), and Turkey (12–14), while in Iraq only few articles have been published; mainly focusing on AIDS/HIV or a specific point related to STIs rather than the subject as a whole (15–18).

This study was done to identify knowledge gaps and negative attitudes toward sexually transmitted infections and infected individuals among undergraduate students. The goal is to give recommendations for the development of more objective research-guided health campaigns and school sex education programs.

## Materials and methods

### Study design and sampling

A cross-sectional study was conducted from May 17, 2022, to June 2, 2022, among undergraduate college students in Iraq using a self-administrated structured questionnaire (Appendix A). The questionnaire was distributed using a web-based Google form across social media websites and applications belonging to public and private universities based in Baghdad, as listed by the Ministry of Higher Education. An explanation of the targeted sample was provided at the top of the questionnaire and on the related website posts, and private social media forums were selected during questionnaire distribution. This was done to reduce the participation of individuals not meeting the sampling criteria—although complete elimination of this bias cannot be achieved with online data collection.

College students were selected as the target sample for two reasons. First, they form a part of the youth high-risk group which tends to have a disproportionally high STI incidence (3) and because they have group-specific risk factors as university, in Iraq, is a period of return to mixed-gender education after 6 years of segregation and therefore more chances for high-risk sexual behavior to occur. Knowledge gaps and attitudes need to be assessed for this group to design targeted public health campaigns and sex education programs.

To increase the validity of our data; First, the research team did not offer any incentives to the respondents to fill out the questionnaire. Second, all questions were mandatory to reduce the number of missing values during the following data analysis steps. Finally, to ensure that no initial data analysis would occur before the end of the data collection period, we made sure that the form would close automatically after 1,000 responses were collected. This was achieved using a Google workplace application, known as “form limiter.” All this was conducted and reported according to the (CHERRIES) checklist for E-surveys (19).

To be included in the final sample, respondents had to fulfill 2 criteria; be enrolled as an undergraduate in a Baghdad-based university during the academic year of 2021/2022 (assigned as criteria 1) and be enrolled in a discipline not related to medicine or medical technology (assigned as criteria 2). This was judged as a necessary step, as previous studies have demonstrated an obvious difference between non-medical and medical college students (11, 18, 20).

### Data collection tool

The questionnaire was developed after a literature review (9, 12, 21). Culture-specific items were then added, including polygamy (described as a man having more than one wife as it is legal in Iraq) and circumcision. The questionnaire was then translated into Arabic and pretested on a small sample of 25 medical students and was subjected to expert review by the department of dermatology at Baghdad Teaching Hospital. Candidiasis was initially included as an item similar to a study from Kampala, Uganda (21). It was removed later as a review revealed that candidiasis is connected to sexual activity itself rather than high-risk behavior or sexual transmission (22, 23).

The questionnaire (Appendix A) was divided into demographics, knowledge, attitudes, and practices. Each section is further divided into blocks with items related to a single topic; for example, within the knowledge section, there were blocks pertaining to diseases, symptoms, transmission, outcomes, sources of information, risk, and protective factors.

In total, there were 84 items related to sexually transmitted infections. Most were presented as Yes/No questions. Knowledge-related items were each assigned 1 point for a total of 60 points from which respondents' overall knowledge could be extrapolated; with those who answered >50% of questions correctly, regarded as having good knowledge. Items related to attitudes and practices, on the other hand, had no similar scoring. This was due to their innate heterogeneity compared to knowledge-related items, it was therefore judged to be more beneficial and representative to discuss each item or block of items separately instead of calculating an overall score.

### Measurements and analysis plan

Statistical Package for the Social Sciences (SPSS) version 24 was used to perform both descriptive measurements (means and proportions) and statistical analysis with an independent-samples *T*-test to assess the effect of gender, knowing someone with an STI, and previous sexual experience on the overall knowledge score. A Chi-square test was also performed to assess the effect of the three factors on each item. Spearman's correlation coefficient was also used to assess the congruency of knowledge self-evaluation with the measured knowledge score. D'Agostino-Pearson-Omnibus test was performed using an Excel statistics plugin as it could not be performed on SPSS.

### Ethical approval and informed consent

A written description of the study's purpose was provided at the top of the questionnaire with information regarding the purpose of the study, the targeted population, and the attainment of study participants' full anonymity during the process of data collection, manuscript writing, and publication. Also, individuals were told on two instances (at the top of the questionnaire and again near the submit button) that by submitting their answers they consent to the usage of the provided information for research which might also include sharing of research data to a data repository to increase transparency. Initial ethical approval was obtained from University of Baghdad/College of Medicine followed by approval from the research committee of the National Center for Training and Human Development belonging to the Ministry of Health and Environment with decision number 8 on May 15, 2022 prior to data collection.

## Results

### Overview

A total of 1,000 responses were collected; 83 individuals did not meet Criteria 1 (not enrolled as undergraduates in a Baghdad-based college) and were therefore excluded from the final sample selection. A further 94 who met Criteria 1 were also excluded because they did not meet Criteria 2 (not enrolled in a medical or a medical technology-related field), providing 823 as the final sample size with a valid response rate of 82.3%.

687 (83.4%) of respondents were equal to or below the age of 24 years and are therefore part of the 15–24 youth group (Table 1). The number of individuals with previous sexual experience is 226 (27.5%), more than twice that of individuals with marital experience (110, 13.4%). The number of individuals with previous sexual but no marital experience is 150 (18.2% of the study sample).

**Table 1:** Sample socio-demographic characteristics.

**Characteristics**	***N* = 823 (%)**
**Age (Years)**	
18–19	130 (15.8)
20–21	273 (33.2)
22–23	249 (30.2)
24–25	73 (8.9)
>25	98 (11.9)
**Gender**	
Male	332 (40.3)
Female	491 (59.7)
**Residency**	
Urban	747 (90.8)
Rural	76 (9.2)
**Marital experience**	
Married/Divorced/Widow	110 (13.4)
No marital experience	713 (86.6)
**Field of study**	
Engineering and technology	249 (30.3)
Education	203 (24.7)
Science studies	165 (20)
Law	64 (7.8)
Business and finances	45 (5.5)
Language	36 (4.4)
Social studies	31 (3.8)
Media and public affairs	18 (2.2)
Others (Arts, Sports, tourism, Etc.)	12 (1.3)
**Do you know someone who has been diagnosed with an STI?**	
Yes	195 (23.7)
No	628 (76.3)
**Previous sexual experience (of any type)**	
Yes	226 (27.5)
No	597 (72.5)

### Overall knowledge

**Figure 1:**
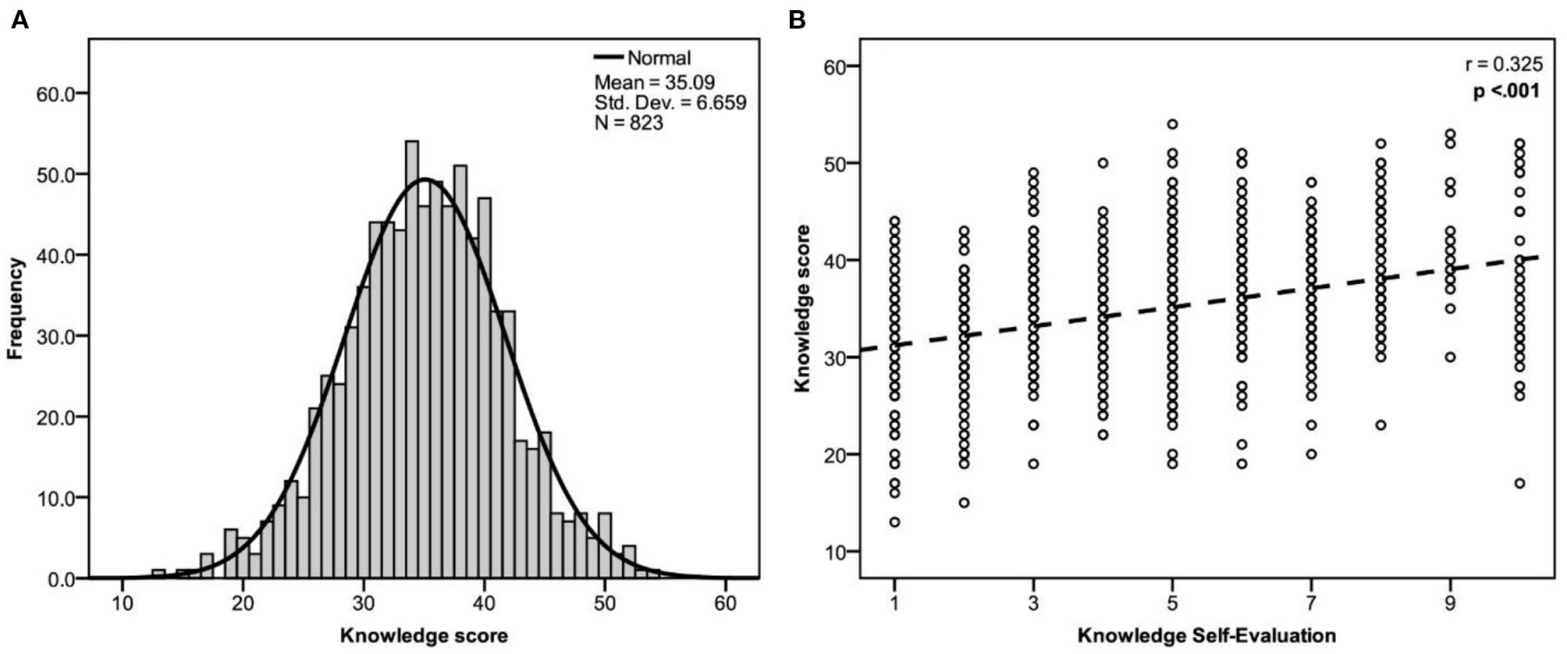
**(A)** Knowledge score distribution, **(B)** Correlation between measured knowledge score and knowledge self-evaluation using spearman's correlation coefficient (r) and a *p*-value of 0.05 as a cutoff point for significance.

As per the Agostino-Pearson-Omnibus test (K^2^ = 1.367, *p* = 0.504), respondents' knowledge scores are normally distributed with a range from 13 to 54, a mean of 35.09 (SD = 6.65) and a median of 35 (IQR = 31–40) (Figure 1). 195 (23.7%) of respondents answered less than half the assigned questions correctly (have a score of < 31). The (B) portion of the figure also shows the relation between the measured (objective) knowledge score and knowledge self-evaluation. Spearman's coefficient (r) indicates a weak but statistically significant correlation measuring 0.325.

Appendix B shows the effect of three factors: gender, previous sexual experience, and knowing someone who has been diagnosed with a sexually transmitted infection; out of these, the latter had the strongest effect, with a mean difference of 2.73 (*P* < 0.001).

### Knowledge of sexually transmitted infections

In our study, the awareness that STIs can be transmitted by sexual intercourse was the most correctly answered item across all categories with a correct response rate of 98.4% (Table 2), closely followed by the awareness of HIV as an STI at 98.2%, while the lowest was the common misconception that showering before and after sex is a protective factor with only 7.9% of the study respondents having the right answer as shown in Appendix B. There were also 21 additional items with > 50% incorrect response rates, these were regarded as “common misconceptions” (Appendix Table B2). Other items exist with < 50% incorrect response rate, but they relate to topics of high public health interest and should therefore be mentioned. These include: the availability of HIV vaccination (standing at 42% incorrect response rate), the non-curability of HIV infections (23.9%), the perception that hormonal contraceptives are useful against STIs (39.2%), and that condoms cause infertility (22.2%).

**Table 2:** Knowledge about sexually transmitted infections among non-medical undergraduates in Baghdad, Iraq.

**Category**	**Gender**			**Do you know someone who has been diagnosed with an STI?**			**Previous sexual experience**		
	**Male 332 (%)^a^**	**Female** **491 (%)^a^**	***p*-value^b^**	**Yes** **195 (%)^a^**	**No 628 (%)^a^**	***p*-value^b^**	**Yes 226 (%)^a^**	**No** **597 (%)^a^**	***p*-value^b^**
**Diseases**									
HIV	326 (98.2)	482 (98.2)	0.978	189 (96.9)	619 (98.6)	0.134	220 (97.3)	588 (98.5)	0.272
Syphilis	124 (37.3)	156 (31.8)	0.098	89 (42.6)	197 (31.4)	**0.004**	91 (40.3)	189 (31.7)	**0.020**
Gonorrhea	224 (67.5)	254 (51.7)	**7** ^*^10^**−6**^	138 (70.8)	340 (54.1)	**5** ^*^10^**−5**^	160 (70.8)	318 (53.3)	**5** ^*^10^**−6**^
Genital warts	83 (25.0)	144 (29.3)	0.173	67 (34.3)	160 (25.5)	**0.015**	80 (35.4)	147 (24.6)	**0.002**
Genital herpes	154 (46.4)	243 (49.5)	0.382	106 (54.4)	291 (46.3)	0.050	110 (48.7)	287 (48.1)	0.878
Chlamydia	84 (25.3)	117 (23.8)	0.630	59 (30.3)	142 (22.6)	**0.030**	65 (28.8)	136 (22.8)	0.075
Trichomoniasis	65 (19.6)	106 (21.6)	0.486	51 (26.2)	120 (19.1)	**0.034**	52 (23.0)	119 (19.9)	0.332
Molluscum	97 (29.2)	136 (27.7)	0.635	77 (39.5)	156 (24.8)	**1** ^*^10^**−4**^	73 (32.3)	160 (26.8)	0.118
Scabies and pediculosis	144 (43.3)	229 (46.6)	0.356	108 (55.4)	265 (42.2)	**0.001**	101 (44.7)	272 (45.6)	0.823
Hepatitis B and C	160 (48.2)	243 (49.5)	0.715	108 (55.4)	295 (47.0)	**0.040**	108 (47.8)	295 (49.4)	0.677
**Symptoms**									
Groin swelling	168 (50.6)	258 (52.5)	0.584	119 (61.0)	307 (48.9)	**0.003**	127 (56.2)	299 (50.1)	0.117
Genital ulcers	247 (74.4)	414 (84.3)	**6** ^*^10^**−4**^	170 (87.2)	491 (78.2)	**0.006**	183 (81.0)	478 (80.1)	0.770
Genital itching	235 (70.8)	366 (74.5)	0.233	157 (80.5)	444 (70.7)	**0.007**	184 (81.4)	417 (69.8)	**0.001**
Genital rash	241 (72.6)	369 (75.2)	0.410	156 (80.0)	454 (72.3)	**0.032**	175 (77.4)	435 (72.9)	0.182
Groin pain	172 (51.8)	290 (59.1)	**0.040**	128 (65.6)	334 (53.2)	**0.012**	128 (56.6)	334 (55.9)	0.859
Painful urination	203 (61.1)	282 (57.4)	0.288	121 (62.1)	364 (58.0)	0.311	140 (61.9)	345 (57.8)	0.279
Menstrual issues	179 (53.9)	257 (52.3)	0.657	112 (57.4)	324 (51.6)	0.153	127 (56.2)	309 (51.8)	0.255
Vaginal discharge	214 (64.5)	335 (68.2)	0.260	142 (72.8)	407 (64.8)	**0.038**	159 (70.4)	390 (65.3)	0.172
Urethral discharge	195 (58.7)	303 (61.7)	0.392	133 (68.2)	365 (58.1)	**0.002**	140 (61.9)	358 (60.0)	0.604
Body rash	135 (40.7)	202 (41.1)	0.891	87 (44.6)	250 (39.8)	0.233	93 (41.2)	244 (40.9)	0.942
Fever	140 (42.2)	217 (44.2)	0.565	104 (53.3)	253 (40.3)	**0.001**	98 (43.4)	259 (43.4)	0.996
Frequent diarrhea	96 (28.9)	127 (25.9)	0.334	59 (30.3)	164 (26.1)	0.256	57 (25.2)	166 (27.8)	0.457
Frequent coughing	78 (23.5)	92 (18.7)	0.098	49 (25.1)	121 (19.3)	0.077	48 (21.2)	122 (20.4)	0.799
Frequent sore throat	66 (19.9)	90 (18.3)	0.564	42 (21.5)	114 (18.2)	0.296	44 (19.6)	112 (18.8)	0.795
Weight loss	122 (36.7)	187 (38.1)	0.697	80 (41.0)	229 (36.5)	0.251	78 (34.5)	231 (38.7)	0.269
No symptoms	275 (82.8)	397 (80.9)	0.472	167 (85.6)	505 (80.4)	0.099	187 (82.7)	485 (81.2)	0.619
**Transmission**									
Sexual intercourse	325 (97.9)	485 (98.8)	0.317	191 (97.9)	619 (98.6)	0.545	222 (98.2)	588 (98.5)	0.788
Skin contact	125 (37.7)	198 (40.3)	0.441	96 (49.2)	227 (36.1)	**0.001**	98 (43.4)	225 (37.7)	0.137
Sharing objects	192 (57.8)	369 (75.2)	**1** ^*^10^**−7**^	144 (73.8)	417 (66.4)	0.051	153 (67.7)	408 (68.3)	0.860
Sharing food^**c**^	166 (50.0)	196 (39.9)	**0.004**	110 (56.4)	252 (40.1)	**8** ^*^10^**−5**^	103 (45.6)	259 (43.4)	0.572
Swimming pools^**c**^	230 (69.3)	415 (84.5)	**1** ^*^10^**−7**^	163 (83.6)	482 (76.8)	**0.043**	180 (79.6)	465 (77.9)	0.585
Blood and injections	304 (91.6)	430 (87.6)	0.071	181 (92.8)	553 (88.1)	0.061	203 (89.8)	531 (88.9)	0.717
Hairdressing	243 (73.2)	378 (77.0)	0.215	164 (84.1)	457 (72.8)	**0.001**	177 (78.3)	444 (74.4)	0.240
Pregnancy and childbirth	184 (55.4)	240 (48.9)	0.065	118 (60.5)	306 (48.7)	**0.004**	131 (58.0)	293 (49.1)	**0.023**
Breastfeeding	174 (52.4)	180 (36.7)	**7** ^*^10^**−6**^	101 (51.8)	253 (40.3)	**0.005**	110 (48.7)	244 (40.9)	**0.044**
Mosquito bite^**c**^	203 (61.1)	281 (57.2)	0.263	130 (66.7)	354 (56.4)	**0.011**	143 (63.3)	341 (57.1)	0.109
**Risk factors**									
Multiple partners	324 (97.6)	476 (96.9)	0.582	190 (97.4)	610 (97.1)	0.823	220 (97.3)	580 (97.2)	0.881
Unprotected sex	300 (90.4)	386 (78.6)	**9** ^*^10^**−6**^	169 (86.7)	517 (82.3)	0.155	190 (84.1)	496 (83.1)	0.734
Substance use	222 (66.9)	357 (72.7)	0.072	145 (74.4)	434 (69.1)	0.161	148 (65.5)	431 (72.2)	0.060
Prostitution	311 (93.7)	452 (92.1)	0.381	184 (94.4)	579 (92.2)	0.310	213 (94.2)	550 (92.1)	0.296
STI co-infection	309 (93.1)	452 (92.1)	0.588	187 (95.9)	574 (91.4)	**0.038**	211 (93.4)	550 (92.1)	0.549
Multiple marriages	180 (54.2)	396 (80.7)	**4** ^*^10^**−16**^	148 (75.9)	428 (68.2)	**0.039**	155 (68.6)	136 (22.8)	0.589
**Prevention**									
Abstinence^**d**^	155 (46.7)	230 (46.8)	0.965	95 (48.7)	290 (46.2)	0.535	93 (41.2)	292 (48.9)	**0.046**
Condoms	287 (86.4)	371 (75.6)	**1** ^*^10^**−4**^	165 (84.6)	493 (78.5)	0.063	192 (85.0)	466 (78.1)	**0.027**
Single partner	268 (80.7)	455 (92.7)	**2** ^*^10^**−7**^	168 (86.2)	555 (88.4)	0.407	190 (84.1)	533 (89.3)	**0.041**
Routine check-up	307 (92.5)	477 (97.1)	**0.002**	187 (95.9)	597 (95.1)	0.632	212 (93.8)	572 (95.8)	0.226
Vaccines (warts)	278 (83.7)	437 (89.0)	**0.028**	169 (86.7)	546 (86.9)	0.921	197 (87.2)	518 (86.8)	0.879
Vaccines (HIV)^**c**^	120 (36.1)	226 (46.0)	**0.005**	95 (48.7)	251 (40.0)	**0.031**	98 (43.4)	248 (41.5)	0.637
Showering before and after sex^**c**^	301 (90.7)	457 (93.1)	0.208	181 (92.8)	577 (91.9)	0.670	208 (92.0)	550 (92.1)	0.965
Contraceptive pill^**c**^	130 (39.2)	193 (39.3)	0.965	90 (46.2)	233 (37.1)	**0.024**	88 (38.9)	235 (39.4)	0.911
Circumcision	238 (71.7)	356 (72.5)	0.797	140 (71.8)	454 (72.3)	0.892	164 (72.6)	430 (72.0)	0.877
**Outcome**									
Resolution (HIV)^**c**^	83 (25.0)	114 (23.2)	0.557	58 (29.7)	139 (22.1)	**0.030**	70 (31.0)	127 (21.3)	**0.004**
Resolution (others)^**c**^	214 (64.5)	285 (58.0)	0.065	127 (65.1)	372 (59.2)	0.141	139 (61.5)	360 (60.3)	0.753
Infertility	178 (53.6)	262 (53.4)	0.943	107 (54.9)	333 (53.0)	0.652	125 (55.3)	315 (52.8)	0.513
Abortion	198 (59.6)	326 (66.4)	**0.048**	133 (68.2)	391 (62.3)	0.132	143 (63.3)	381 (63.8)	0.885
Premature birth	149 (44.9)	254 (51.7)	0.054	99 (50.8)	304 (48.4)	0.564	126 (55.8)	277 (46.4)	**0.017**
Birth defects	201 (60.5)	303 (61.7)	0.736	123 (63.1)	381 (60.7)	0.547	143 (63.3)	361 (60.5)	0.461
Kidney problems	260 (78.3)	361 (73.5)	0.117	163 (83.6)	458 (72.9)	**0.003**	177 (78.3)	444 (74.4)	0.240
Cancer	193 (58.1)	313 (63.7)	0.104	127 (65.1)	379 (60.4)	0.231	140 (61.9)	366 (61.3)	0.866
Death	219 (66.0)	318 (64.8)	0.723	137 (70.3)	400 (63.7)	0.093	143 (63.3)	394 (66.0)	0.464
**Information source**									
School	170 (51.2)	249 (50.7)	0.890	95 (48.7)	324 (51.6)	0.483	111 (49.1)	308 (51.6)	0.526
Healthcare providers	148 (44.6)	194 (39.5)	0.148	99 (50.8)	243 (38.7)	**0.003**	104 (46.0)	238 (39.9)	0.110
Parents	93 (28.0)	159 (32.4)	0.182	75 (38.5)	177 (28.2)	**0.007**	69 (30.5)	183 (30.7)	0.973
Friends	216 (65.1)	212 (43.2)	**7** ^*^10^**−10**^	121 (62.1)	307 (48.9)	**0.001**	146 (64.6)	282 (47.2)	**8** ^*^10^**−6**^
Books	207 (62.3)	279 (56.8)	0.114	124 (63.6)	362 (57.6)	0.140	154 (68.1)	332 (55.6)	**0.001**
TV	188 (56.6)	270 (55.0)	0.643	111 (56.9)	347 (55.3)	0.682	134 (59.3)	324 (54.3)	0.196
The Internet	313 (94.6)	451 (91.9)	0.137	183 (93.8)	581 (92.7)	0.573	211 (93.8)	553 (92.6)	0.567

^a^Counts and column percent are described as individuals who have answered “Yes” during data collection.

^b^Chi-square for association with a cutoff point of 0.05 for p-value and significant results indicated with a bold text.

^c^For these questions, “No” was the correct answer.

^d^During data collection, abstinence was described as restraining from sexual experience before marriage.

### Attitudes toward sexually transmitted infections

82.6% of respondents agreed that sex education should be taught in middle or high school (Table 3). For these individuals, “traditional barriers” was selected as the most important barrier against the implementation of such programs (64.77%), followed by the sensitivity of the subject (24.57%) and religious barriers (10.65%) (Appendix C). This distribution was different for individuals who were against teaching sex education. On average, individuals who agreed to sex education scored 2.3 points higher on the knowledge scale (*P* < 0.001).

Table 3Attitudes toward sexually transmitted infections, their prevention, and infected individuals among non-medical undergraduates in Baghdad, Iraq.

**Table:** 

**Categories**	**Gender**			**Do you know someone who has been diagnosed with an** **STI?**		
	**Male 332 (%)** ^a^	**Female 491 (%)** ^a^	***P*** **-value** ^b^	**Yes 195 (%)** ^a^	**No 628 (%)** ^a^	***P*** **-value** ^b^
**Sexually transmitted infections**						
Can be effectively prevented	317 (95.5)	472 (96.1)	0.647	187 (95.9)	602 (95.9)	0.982
**Public health campaigns**						
Have made you reconsider sex	270 (81.3)	410 (83.5)	0.419	166 (85.1)	514 (81.8)	0.291
More campaigns are needed	319 (96.1)	474 (96.5)	0.734	189 (96.9)	604 (96.2)	0.628
**Sex education**						
Should be taught in middle/high school	280 (84.3)	424 (86.4)	0.420	173 (88.7)	531 (84.6)	0.291
Should be a part of science class	269 (81.0)	429 (87.4)	**0.013**	164 (84.1)	534 (85.0)	0.628
**Condoms**						
Can cause infertility	62 (18.7)	121 (24.6)	**0.043**	48 (24.6)	135 (21.5)	0.360
Can increase participation in casual sex	177 (53.3)	317 (64.6)	**0.001**	108 (55.4)	386 (61.5)	0.130
Can decrease sexual pleasure	280 (84.3)	310 (63.1)	**3** ^*^ **10** ^**∧**^ **−11**	149 (76.4)	441 (70.2)	0.094
Can lead to partner mistrust	212 (63.9)	259 (52.7)	**0.002**	114 (58.5)	357 (56.8)	0.691
Are not effective when used as the only infection prevention method	188 (56.6)	351 (71.5)	**1** ^*^ **10** ^**∧**^ **−5**	125 (64.1)	414 (65.9)	0.640
**Individuals with STIs**						
Should be socially isolated	211 (63.6)	288 (58.7)	0.158	120 (61.5)	379 (60.4)	0.767
Should suffer from violence	114 (34.3)	147 (29.9)	0.183	61 (31.3)	200 (31.8)	0.882
Should have fewer jobs	156 (47.0)	178 (36.3)	**0.002**	84 (43.1)	250 (39.8)	0.417
Should be stigmatized by doctors	171 (51.5)	222 (45.2)	0.076	95 (48.7)	298 (47.5)	0.757

**Table:** 

**Categories**	**Previous sexual experience**		
	**Yes 226 (%)** ^a^	**No 597 (%)** ^a^	***P*** **-value** ^b^
**Sexually transmitted infections**			
Can be effectively prevented	219 (96.9)	570 (95.5)	0.359
**Public health campaigns**			
Have made you reconsider sex	186 (82.3)	494 (82.7)	0.880
More campaigns are needed	218 (96.5)	575 (96.3)	0.921
**Sex education**			
Should be taught in middle/high school	196 (86.7)	508 (85.1)	0.552
Should be a part of science class	191 (84.5)	507 (84.9)	0.883
**Condoms**			
Can cause infertility	45 (19.9)	138 (23.1)	0.324
Can increase participation in casual sex	111 (49.1)	383 (64.2)	**8** ^*^ **10** ^**∧**^ **−5**
Can decrease sexual pleasure	175 (77.4)	415 (69.5)	**0.024**
Can lead to partner mistrust	117 (51.8)	354 (59.3)	0.051
Are not effective when used as the only infection prevention method	139 (61.5)	400 (67.0)	0.139
**Individuals with STIs**			
Should be socially isolated	137 (60.6)	562 (60.6)	0.996
Should suffer from violence	71 (31.4)	190 (31.8)	0.910
Should have fewer jobs	87 (38.5)	247 (41.4)	0.453
Should be stigmatized by doctors	105 (46.5)	288 (48.2)	0.648

a Counts and column percent are described as individuals who have answered “Yes” during data collection.

b Chi-square for association with a cutoff point of 0.05 for p-value and significant results indicated with a bold text.

### Practices related to sexually transmitted infections

Individuals who had a previous sexual experience were 2 years older on average and more likely to be men (41.3% of men had some sort of sexual experience compared to 18.1% of women) and as demonstrated in Appendix D, they were also more likely to know someone who has been diagnosed with an STI compared to those without sexual experience. Other practices related to suspicion or after a diagnosis with an STI are summarized in Table 4.

Table 4Practices upon suspicion or diagnosis with a sexually transmitted infection among non-medical undergraduates in Baghdad, Iraq.

**Table:** 

**Categories**	**Gender**			**Do you know someone who has been diagnosed with an STI?**		
	**Male 332 (%)** ^a^	**Female 491 (%)** ^a^	***P*** **-value** ^b^	**Yes 195 (%)** ^a^	**No 628 (%)** ^a^	***P*** **-value** ^b^
**Suspicion of having an STI due to symptoms or after high-risk behavior**						
Ask your parent	129 (38.9)	198 (40.3)	0.672	77 (39.5)	250 (39.8)	0.936
Ask a friend	149 (44.9)	158 (32.2)	**8** ^*^ **10** ^**∧**^ **−5**	76 (39.0)	231 (36.8)	0.581
Seek medical advice	317 (95.5)	449 (91.4)	**0.025**	181 (92.8)	585 (93.2)	0.873
Search the internet	295 (88.9)	447 (91.0)	0.302	176 (90.3)	566 (90.1)	0.958
Ignore this suspicion if no symptoms	111 (33.4)	145 (29.5)	0.235	59 (30.3)	197 (31.4)	0.769
**Diagnosis with an STI**						
Follow the doctor's advice	320 (96.4)	476 (96.9)	0.658	187 (95.9)	609 (97.0)	0.461
Self-medicate with OTC drugs^c^	55 (16.6)	91 (18.5)	0.469	45 (23.1)	101 (16.1)	**0.026**
Seek herbal and traditional medicine	105 (31.6)	133 (27.1)	0.159	63 (32.3)	175 (27.9)	0.232
Ignore the diagnosis if mild	48 (14.5)	82 (16.7)	0.387	38 (19.5)	92 (14.6)	0.106

**Table:** 

**Categories**	**Previous sexual experience**		
	**Yes 226 (%)** ^a^	**No 597 (%)** ^a^	***p*** **-value** ^b^
**Suspicion of having an STI due to symptoms or after high-risk behavior**			
Ask your parent	72 (31.9)	255 (42.7)	**0.005**
Ask a friend	93 (41.2)	214 (35.8)	0.160
Seek medical advice	213 (94.2)	553 (92.6)	0.415
Search the internet	203 (89.8)	539 (90.3)	0.843
Ignore this suspicion if no symptoms	67 (29.6)	189 (31.7)	0.578
**Diagnosis with an STI**			
Follow the doctor's advice	217 (96.0)	579 (97.0)	0.487
Self-medicate with OTC drugs^c^	45 (19.9)	101 (16.9)	0.316
Seek herbal and traditional medicine	64 (28.3)	174 (29.1)	0.815
Ignore the diagnosis if mild	35 (15.5)	95 (15.9)	0.881

a Counts and column percent are described as individuals who have answered “Yes” during data collection.

b Chi-square for association with a cutoff point of 0.05 for p-value and significant results indicated with a bold text.

c OTC, over the counter.

## Discussion

The current study revealed that overall knowledge was moderate to high, with around three-quarters of respondents answering more than half the questions correctly; this is higher than the overall knowledge reported in other studies from Kufa in south Iraq (18), Iran (9), and Saudi Arabia (11) and comparable to results from Malaysia (20, 24). In this study, knowledge about STIs was not affected by gender. This is similar to several studies (9, 20, 24, 25), but differs from two studies in which women had higher knowledge (11, 18). Knowing someone who has been infected with an STI was more critical to increase knowledge than having a previous sexual experience. This is a concerning finding, as knowledge is acquired after someone else has already been affected. It also shows that people who might be thinking about having a sexual relationship might not have access to better sources of information, making school-based sex education programs and targeted public health campaigns more essential.

Internet was the most common source of information, as with most studies (9, 10, 20). However, this was not the case in one Turkish study from 2014 in which written media like books, magazines, and newspapers took first place (13). Studies on the credibility of Arab health-related websites show that only a minority of the websites are credible and certified by the Health on the Net (HON) foundation (26, 27). Social media is probably more problematic but less investigated. This dependence on online information may stem from the void created by a lack of sufficient information from other sources which were less cited (9, 10, 13). Furthermore, almost all the respondents think that more public health campaigns are needed and more than 80% think that sex education should be taught in middle or high school. This propensity to know more about STIs must be met with credible sources under the supervision of Iraqi health and education authorities.

HIV was the most commonly recognized STI (98.2%). The same finding was obtained from other research (8, 10, 12–14, 20, 24). A study conducted on Iraqi females published in 2008 showed that only half the respondents had heard about HIV/AIDS and 88.5% recognized sex as a transmission route (17). Another study conducted on high school students in Erbil, north of Iraq, in 2015 showed a higher level of recognition for HIV sexual transmission (94.3%) (16). It should be noted that both of these studies did not mention systemic manifestations commonly associated with AIDS (frequent diarrhea, frequent cough, and frequent sore throat). In our study, less than half the respondents recognized these as symptoms of STIs. As for other HIV items, only 58% of respondents correctly identified the unavailability of a vaccine, and 76.1% correctly identified HIV as a non-curable infection. The vaccine misconception was less prominent in two other studies from Iraq focusing on knowledge of HIV in a sample of Erbil high school students and university students from two governorates (15, 16), in the latter those from Diyala were more likely to believe in the vaccine misconception. All of this indicates that although more people now recognize HIV as an STI, there are still gaps in knowledge that might lead to lower risk perception and delayed care-seeking.

In terms of non-HIV STIs, trichomoniasis and chlamydia have the highest incidence among all STIs both globally and in the Middle East/North Africa region (1, 4). Yet, they were the least recognized in this study and many other studies (8, 10, 13, 24). Men were more likely to recognize gonorrhea as an STI similar to another study conducted in Iran (9) since gonorrhea is more symptomatic in men (28). In contrast, women were more likely to recognize the presence of HPV vaccines, presumably because cervical cancer in women is given more public health attention than HPV outcomes in men. This gender difference was also found in other studies (29–31). Half the respondents did not recognize pregnancy and childbirth as modes of transmission, and an even higher portion did not recognize breastfeeding as such. These are important topics that should be focused on in future educational campaigns as they relate to a significant risk to the baby from severe forms of non-HIV sexually transmitted infections including chronic hepatitis B, gonococcal conjunctivitis, and HSV, along with HIV transmission (28).

An interesting trend is noted regarding gender differences in the recognition of preventive methods; men were more likely to recognize condoms as a protective method, even when used alone, and unprotected sex as a risk factor, making men favor protection over monogamy. Women, on the other hand, were more likely to recognize polygamy (which is permissible in the Muslim world) as a risk factor, and having a single partner as a protective factor, which indicates a propensity for monogamy as a preventive method. This notion was not found in a study from Iran (9) where females were more likely to cite condom non-use as a risk factor.

Although there was relatively high support for school sex education programs, individuals who did not support such measures reported sensitivity and religious barriers more frequently. This means that future programs need to be modified and modulated in a generally acceptable way to traverse these two barriers. Still, the most cited barrier overall was traditional barriers, exemplified by tribalism which constitutes a part of legal pluralism in Iraq (32). This is unlikely to be solved just by adjusting the content of sex education programs and might need a wider governmental approach outside the scope of sexual health education. The notion that higher overall knowledge correlated with higher acceptance of sex education programs points out that it might also be possible to increase acceptance by improving knowledge from other sources like the media, the internet, or by community-based public health campaigns, and not only by addressing the aforementioned barriers.

Despite the moderately good level of knowledge, attitudes toward infected individuals were still negative, especially social stigmatization. People in favor of the social isolation of infected individuals were more likely to recognize sharing food and swimming pools as possible transmission routes for STIs (Appendix C). Women were more empathetic in terms of institutional stigmatization. This might be explained by women having a more compassionate nature than men (33) or due to socio-demographic differences between the two genders locally (34).

A significant portion of respondents, especially men and individuals with previous sexual experience, had the attitude that condoms decrease sexual pleasure. While women and those without sexual experience were more likely to think that condoms can increase participation in casual sex. These concerns should be addressed in future educational programs if an attempt is to be made to encourage condom usage. A previous study done in north Iraq also showed that an increased individual's risk perception was associated with more condom use, which may indicate that increasing overall knowledge about STIs might also increase condom use (35).

College students were more likely to have sex as they got older. This can be explained by increased independence from parents and is supported by the lower likelihood of asking parents for advice when suspecting being infected. This means that if sex education was to be implemented in college, implementing it in early grades would be more beneficial. Women were less likely to have had sex, similar to another study (14), and less likely to ask friends for advice upon suspicion of having an STI, largely because females are more stigmatized in terms of sexual activity and even more in the context of STIs (36). This stigma may also delay or prevent test seeking (37). People who know someone with an STI are more likely to self-medicate with over-the-counter drugs on suspicion of having an STI. This might be explained by studies done on self-medication behavior in Iraq and the Middle East, in which family and friends were prominent sources for self-medication advice (38, 39).

### Limitations

The main shortcoming of this study is that the sample was convenient and not random. This is due to the lack of an official body to facilitate the random allocation of university students. In addition, the heterogeneity of STIs, especially in terms of their transmission routes, might call for separate studies for each individual disease. Finally, although an explanation of the targeted sample was provided at the top of the questionnaire and on the related website posts, and the questionnaire was distributed to private social media forums, belonging to Baghdad-based universities, to decrease the participation of individuals who don't meet the sample criteria, online data collection methodically leads to an inability to verify the integrity of participants' answers or sample selection.

## Conclusion and recommendations

Despite the good level of recognition for HIV and gonorrhea as sexually transmitted infections, certain knowledge gaps still persist for both HIV (recognizing systemic symptoms usually associated with AIDS, unavailability of a vaccine, and non-curability of HIV infections) and non-HIV infections (recognizing chlamydia and trichomoniasis as STIs and vertical transmission). Most students showed openness toward public health campaigns and school sex education, but negative attitudes were still found toward condom usage and STI-infected individuals with certain gender variations. All the aforementioned points should be addressed in any future sex education program or public health campaign. Such interventions should be started early before sexual debut and modified according to the needs of each demographic group.

## Data availability statement

The original contributions presented in the study are publicly available. This data can be found here: https://data.mendeley.com/datasets/2dmhrmt7n8/3.

## Ethics statement

The studies involving human participants were reviewed and approved by the Research Committee of the National Center for Training and Human Development belonging to the Ministry of Health and Environment. The patients/participants provided their written informed consent to participate in this study.

## Author contributions

JA-D and AA-S led the conception of the research idea. GA-G, AA-S, and JA-D designed the data collection tool and participated in data collection. FL reviewed the study design and reviewed the manuscript for intellectual content. JA-D applied for and provided ethical approvals for the implementation of the study. GA-G led the data analysis. AA-S and GA-G interpreted the data. All authors drafted the manuscript, attain full responsibility for the accuracy, integrity of the work, and have approved the final version of the manuscript.
