# Corrigendum: Assessing knowledge, attitudes, and practices toward sexually transmitted infections among Baghdad undergraduate students for research-guided sexual health education

**DOI:** 10.3389/fpubh.2025.1617766

**Published:** 2025-06-24

**Authors:** Ghaith Al-Gburi, Ali Al-Shakarchi, Jaafar D. Al-Dabagh, Faris Lami

**Affiliations:** ^1^College of Medicine, University of Baghdad, Baghdad, Iraq; ^2^Department of Community and Family Medicine, College of Medicine, University of Baghdad, Baghdad, Iraq

**Keywords:** sexually transmitted infections, sex education, Middle East, Iraq, Baghdad

In the published article, there was an error. The proportion of participants who believed that HIV could be completely cured was reported as 32.9% in the text instead of the correct figure of 23.9%, which could be derived from Table 2. A correction has been made to **[Results]**, *[Knowledge of sexually transmitted infections*]. This sentence previously stated:

“These include: the availability of HIV vaccination (standing at 42% incorrect response rate), the non-curability of HIV infections (32.9%), …”

The corrected sentence appears below:

“These include: the availability of HIV vaccination (standing at 42% incorrect response rate), the non-curability of HIV infections (23.9%), …”

In the published article, there was an error. The proportion of participants who believed that HIV is a non-curable infection was reported as 67.1% in the text instead of 76.1%, which could be derived from Table 2.

A correction has been made to the **[Discussion]**, Paragraph 3. This sentence previously stated:

“As for other HIV items, only 58% of respondents correctly identified the unavailability of a vaccine, and 67.1% correctly identified HIV as a non-curable infection.”

The corrected sentence appears below:

“As for other HIV items, only 58% of respondents correctly identified the unavailability of a vaccine, and 76.1% correctly identified HIV as a non-curable infection.”

In the published article, there was an error in Table 2 as published. The “c” superscript denoting “No” as the correct answer was not placed for two items: “Showering before and after sex” and “contraceptive pill”. The corrected Table 2 and its caption appear below.

**Table 2:** Knowledge about sexually transmitted infections among non-medical undergraduates in Baghdad, Iraq.

**Category**	**Gender**			**Do you know someone who has been diagnosed with an STI?**			**Previous sexual experience**		
	**Male 332 (%)^a^**	**Female** **491 (%)^a^**	***p*-value^b^**	**Yes** **195 (%)^a^**	**No 628 (%)^a^**	***p*-value^b^**	**Yes 226 (%)^a^**	**No** **597 (%)^a^**	***p*-value^b^**
**Diseases**									
HIV	326 (98.2)	482 (98.2)	0.978	189 (96.9)	619 (98.6)	0.134	220 (97.3)	588 (98.5)	0.272
Syphilis	124 (37.3)	156 (31.8)	0.098	89 (42.6)	197 (31.4)	**0.004**	91 (40.3)	189 (31.7)	**0.020**
Gonorrhea	224 (67.5)	254 (51.7)	**7** ^*^10^**−6**^	138 (70.8)	340 (54.1)	**5** ^*^10^**−5**^	160 (70.8)	318 (53.3)	**5** ^*^10^**−6**^
Genital warts	83 (25.0)	144 (29.3)	0.173	67 (34.3)	160 (25.5)	**0.015**	80 (35.4)	147 (24.6)	**0.002**
Genital herpes	154 (46.4)	243 (49.5)	0.382	106 (54.4)	291 (46.3)	0.050	110 (48.7)	287 (48.1)	0.878
Chlamydia	84 (25.3)	117 (23.8)	0.630	59 (30.3)	142 (22.6)	**0.030**	65 (28.8)	136 (22.8)	0.075
Trichomoniasis	65 (19.6)	106 (21.6)	0.486	51 (26.2)	120 (19.1)	**0.034**	52 (23.0)	119 (19.9)	0.332
Molluscum	97 (29.2)	136 (27.7)	0.635	77 (39.5)	156 (24.8)	**1** ^*^10^**−4**^	73 (32.3)	160 (26.8)	0.118
Scabies and pediculosis	144 (43.3)	229 (46.6)	0.356	108 (55.4)	265 (42.2)	**0.001**	101 (44.7)	272 (45.6)	0.823
Hepatitis B and C	160 (48.2)	243 (49.5)	0.715	108 (55.4)	295 (47.0)	**0.040**	108 (47.8)	295 (49.4)	0.677
**Symptoms**									
Groin swelling	168 (50.6)	258 (52.5)	0.584	119 (61.0)	307 (48.9)	**0.003**	127 (56.2)	299 (50.1)	0.117
Genital ulcers	247 (74.4)	414 (84.3)	**6** ^*^10^**−4**^	170 (87.2)	491 (78.2)	**0.006**	183 (81.0)	478 (80.1)	0.770
Genital itching	235 (70.8)	366 (74.5)	0.233	157 (80.5)	444 (70.7)	**0.007**	184 (81.4)	417 (69.8)	**0.001**
Genital rash	241 (72.6)	369 (75.2)	0.410	156 (80.0)	454 (72.3)	**0.032**	175 (77.4)	435 (72.9)	0.182
Groin pain	172 (51.8)	290 (59.1)	**0.040**	128 (65.6)	334 (53.2)	**0.012**	128 (56.6)	334 (55.9)	0.859
Painful urination	203 (61.1)	282 (57.4)	0.288	121 (62.1)	364 (58.0)	0.311	140 (61.9)	345 (57.8)	0.279
Menstrual issues	179 (53.9)	257 (52.3)	0.657	112 (57.4)	324 (51.6)	0.153	127 (56.2)	309 (51.8)	0.255
Vaginal discharge	214 (64.5)	335 (68.2)	0.260	142 (72.8)	407 (64.8)	**0.038**	159 (70.4)	390 (65.3)	0.172
Urethral discharge	195 (58.7)	303 (61.7)	0.392	133 (68.2)	365 (58.1)	**0.002**	140 (61.9)	358 (60.0)	0.604
Body rash	135 (40.7)	202 (41.1)	0.891	87 (44.6)	250 (39.8)	0.233	93 (41.2)	244 (40.9)	0.942
Fever	140 (42.2)	217 (44.2)	0.565	104 (53.3)	253 (40.3)	**0.001**	98 (43.4)	259 (43.4)	0.996
Frequent diarrhea	96 (28.9)	127 (25.9)	0.334	59 (30.3)	164 (26.1)	0.256	57 (25.2)	166 (27.8)	0.457
Frequent coughing	78 (23.5)	92 (18.7)	0.098	49 (25.1)	121 (19.3)	0.077	48 (21.2)	122 (20.4)	0.799
Frequent sore throat	66 (19.9)	90 (18.3)	0.564	42 (21.5)	114 (18.2)	0.296	44 (19.6)	112 (18.8)	0.795
Weight loss	122 (36.7)	187 (38.1)	0.697	80 (41.0)	229 (36.5)	0.251	78 (34.5)	231 (38.7)	0.269
No symptoms	275 (82.8)	397 (80.9)	0.472	167 (85.6)	505 (80.4)	0.099	187 (82.7)	485 (81.2)	0.619
**Transmission**									
Sexual intercourse	325 (97.9)	485 (98.8)	0.317	191 (97.9)	619 (98.6)	0.545	222 (98.2)	588 (98.5)	0.788
Skin contact	125 (37.7)	198 (40.3)	0.441	96 (49.2)	227 (36.1)	**0.001**	98 (43.4)	225 (37.7)	0.137
Sharing objects	192 (57.8)	369 (75.2)	**1** ^*^10^**−7**^	144 (73.8)	417 (66.4)	0.051	153 (67.7)	408 (68.3)	0.860
Sharing food^**c**^	166 (50.0)	196 (39.9)	**0.004**	110 (56.4)	252 (40.1)	**8** ^*^10^**−5**^	103 (45.6)	259 (43.4)	0.572
Swimming pools^**c**^	230 (69.3)	415 (84.5)	**1** ^*^10^**−7**^	163 (83.6)	482 (76.8)	**0.043**	180 (79.6)	465 (77.9)	0.585
Blood and injections	304 (91.6)	430 (87.6)	0.071	181 (92.8)	553 (88.1)	0.061	203 (89.8)	531 (88.9)	0.717
Hairdressing	243 (73.2)	378 (77.0)	0.215	164 (84.1)	457 (72.8)	**0.001**	177 (78.3)	444 (74.4)	0.240
Pregnancy and childbirth	184 (55.4)	240 (48.9)	0.065	118 (60.5)	306 (48.7)	**0.004**	131 (58.0)	293 (49.1)	**0.023**
Breastfeeding	174 (52.4)	180 (36.7)	**7** ^*^10^**−6**^	101 (51.8)	253 (40.3)	**0.005**	110 (48.7)	244 (40.9)	**0.044**
Mosquito bite^**c**^	203 (61.1)	281 (57.2)	0.263	130 (66.7)	354 (56.4)	**0.011**	143 (63.3)	341 (57.1)	0.109
**Risk factors**									
Multiple partners	324 (97.6)	476 (96.9)	0.582	190 (97.4)	610 (97.1)	0.823	220 (97.3)	580 (97.2)	0.881
Unprotected sex	300 (90.4)	386 (78.6)	**9** ^*^10^**−6**^	169 (86.7)	517 (82.3)	0.155	190 (84.1)	496 (83.1)	0.734
Substance use	222 (66.9)	357 (72.7)	0.072	145 (74.4)	434 (69.1)	0.161	148 (65.5)	431 (72.2)	0.060
Prostitution	311 (93.7)	452 (92.1)	0.381	184 (94.4)	579 (92.2)	0.310	213 (94.2)	550 (92.1)	0.296
STI co-infection	309 (93.1)	452 (92.1)	0.588	187 (95.9)	574 (91.4)	**0.038**	211 (93.4)	550 (92.1)	0.549
Multiple marriages	180 (54.2)	396 (80.7)	**4** ^*^10^**−16**^	148 (75.9)	428 (68.2)	**0.039**	155 (68.6)	136 (22.8)	0.589
**Prevention**									
Abstinence^**d**^	155 (46.7)	230 (46.8)	0.965	95 (48.7)	290 (46.2)	0.535	93 (41.2)	292 (48.9)	**0.046**
Condoms	287 (86.4)	371 (75.6)	**1** ^*^10^**−4**^	165 (84.6)	493 (78.5)	0.063	192 (85.0)	466 (78.1)	**0.027**
Single partner	268 (80.7)	455 (92.7)	**2** ^*^10^**−7**^	168 (86.2)	555 (88.4)	0.407	190 (84.1)	533 (89.3)	**0.041**
Routine check-up	307 (92.5)	477 (97.1)	**0.002**	187 (95.9)	597 (95.1)	0.632	212 (93.8)	572 (95.8)	0.226
Vaccines (warts)	278 (83.7)	437 (89.0)	**0.028**	169 (86.7)	546 (86.9)	0.921	197 (87.2)	518 (86.8)	0.879
Vaccines (HIV)^**c**^	120 (36.1)	226 (46.0)	**0.005**	95 (48.7)	251 (40.0)	**0.031**	98 (43.4)	248 (41.5)	0.637
Showering before and after sex^**c**^	301 (90.7)	457 (93.1)	0.208	181 (92.8)	577 (91.9)	0.670	208 (92.0)	550 (92.1)	0.965
Contraceptive pill^**c**^	130 (39.2)	193 (39.3)	0.965	90 (46.2)	233 (37.1)	**0.024**	88 (38.9)	235 (39.4)	0.911
Circumcision	238 (71.7)	356 (72.5)	0.797	140 (71.8)	454 (72.3)	0.892	164 (72.6)	430 (72.0)	0.877
**Outcome**									
Resolution (HIV)^**c**^	83 (25.0)	114 (23.2)	0.557	58 (29.7)	139 (22.1)	**0.030**	70 (31.0)	127 (21.3)	**0.004**
Resolution (others)^**c**^	214 (64.5)	285 (58.0)	0.065	127 (65.1)	372 (59.2)	0.141	139 (61.5)	360 (60.3)	0.753
Infertility	178 (53.6)	262 (53.4)	0.943	107 (54.9)	333 (53.0)	0.652	125 (55.3)	315 (52.8)	0.513
Abortion	198 (59.6)	326 (66.4)	**0.048**	133 (68.2)	391 (62.3)	0.132	143 (63.3)	381 (63.8)	0.885
Premature birth	149 (44.9)	254 (51.7)	0.054	99 (50.8)	304 (48.4)	0.564	126 (55.8)	277 (46.4)	**0.017**
Birth defects	201 (60.5)	303 (61.7)	0.736	123 (63.1)	381 (60.7)	0.547	143 (63.3)	361 (60.5)	0.461
Kidney problems	260 (78.3)	361 (73.5)	0.117	163 (83.6)	458 (72.9)	**0.003**	177 (78.3)	444 (74.4)	0.240
Cancer	193 (58.1)	313 (63.7)	0.104	127 (65.1)	379 (60.4)	0.231	140 (61.9)	366 (61.3)	0.866
Death	219 (66.0)	318 (64.8)	0.723	137 (70.3)	400 (63.7)	0.093	143 (63.3)	394 (66.0)	0.464
**Information source**									
School	170 (51.2)	249 (50.7)	0.890	95 (48.7)	324 (51.6)	0.483	111 (49.1)	308 (51.6)	0.526
Healthcare providers	148 (44.6)	194 (39.5)	0.148	99 (50.8)	243 (38.7)	**0.003**	104 (46.0)	238 (39.9)	0.110
Parents	93 (28.0)	159 (32.4)	0.182	75 (38.5)	177 (28.2)	**0.007**	69 (30.5)	183 (30.7)	0.973
Friends	216 (65.1)	212 (43.2)	**7** ^*^10^**−10**^	121 (62.1)	307 (48.9)	**0.001**	146 (64.6)	282 (47.2)	**8** ^*^10^**−6**^
Books	207 (62.3)	279 (56.8)	0.114	124 (63.6)	362 (57.6)	0.140	154 (68.1)	332 (55.6)	**0.001**
TV	188 (56.6)	270 (55.0)	0.643	111 (56.9)	347 (55.3)	0.682	134 (59.3)	324 (54.3)	0.196
The Internet	313 (94.6)	451 (91.9)	0.137	183 (93.8)	581 (92.7)	0.573	211 (93.8)	553 (92.6)	0.567

^a^Counts and column percent are described as individuals who have answered “Yes” during data collection.

^b^Chi-square for association with a cutoff point of 0.05 for p-value and significant results indicated with a bold text.

^c^For these questions, “No” was the correct answer.

^d^During data collection, abstinence was described as restraining from sexual experience before marriage.

In the published article, there was missing information. Effect size indices were not reported for Tables B1, C2, D1. This information is now included in Supplementary Table 1. For cases where equality of variance could be assumed, based on Levene's test, the Cohen's D index is reported. Alternatively, the mean difference was scaled by the square root of the average variance (instead of the pooled variance) as a more accurate indicator of the effect size (1). For both indices, a value of ≥0.2 signifies a small effect, ≥0.5 signifies a moderate effect, and ≥0.8 for a large effect.


$$d=\;\frac{{\bar{X}}_{1}-{\bar{X}}_{2}}{S_{pooled}},\;d_{SRMS}=\;\frac{{\bar{X}}_{1}-{\bar{X}}_{2}}{\sqrt{\frac{S_{1}^{2}\;+\;S_{2}^{2}}{2}}}$$

**Supplementary Table 1:** Effect size indices for independent samples T-test (N = 823).

**Scale**	**Categorical variable**	**Groups**	**Mean (SD)**	**Mean difference**	**Levene's Test**	**T-test**	**Effect size**
Knowledge score	Gender	Male	34.82 (±6.8)	0.450	F = 1.359	t = −0.952^**a**^	−0.068^**c**^
		Female	35.27 (±6.5)		p = 0.244	p = 0.341	
	Do you know someone with an STD?	Yes	37.17 (±6.7)	2.730	F = 0.121	t = 5.076^**a**^	0.416^**c**^
		No	34.44 (±6.4)		p = 0.728	p = **4.8** ^*^10^**−7**^	
	Previous sexual experience	Yes	35.77 (±7.0)	0.935	F = 2.317	t = 1.802^**a**^	0.141^**c**^
		No	34.83 (±6.4)		p = 0.128	p = 0.072	
	Sex education should be taught in school	Yes	35.42 (±6.6)	2.307	F = 0.249	t = 3.519^**a**^	0.349^**c**^
		No	33.11 (±6.6)		p = 0.618	p = **4.5** ^*^10^**−4**^	
Age (Years)	Previous sexual experience	Yes	23.74 (±4.7)	2.050	F = 34.015	t = 6.033^**b**^	0.525^**d**^
		No	21.69 (±2.9)		p = **7.9** ^*^10^**−9**^	p = **4.8** ^*^10^**−9**^	

^a^Student's independent samples T-test was used to test for association with 0.05 as a cut-off point for statistical significance.

^b^Welch's independent samples T-test was used to test for association with 0.05 as a cut-off point for statistical significance.

^c^Cohen's D was used to assess effect size with ≥0.2 for small, ≥0.5 for moderate, and ≥0.8 for large effect sizes.

^d^The mean difference scaled by the square root of the average variance was used to assess effect size with ≥0.2 for small, ≥0.5 for moderate, and ≥0.8 for large effect sizes.

In the published article, in Tables 2–4, a large number of hypothesis testing was performed simultaneously to test the association between each item and the three independent variables (gender, knowing someone who has been diagnosed with a sexually transmitted infection, and previous sexual experience). To further strengthen statistical reporting, the Benjamin-Hochberg procedure could be utilized to adjust p-values for multiple testing and reduce the false discovery rate to 5% within each family of hypothesis tests (within each independent variable) (2). This information is now provided in Supplementary Tables 2–4, which also include the odds ratio as an index of effect size to improve the interpretability of the statistically significant associations.

**Supplementary Table 2:** Odds ratios and adjusted p-values for the knowledge about sexually transmitted infections among non-medical undergraduates in Baghdad, Iraq.

**Category**	**Gender**		**Do you know someone who has been diagnosed with an STI?**		**Previous sexual experience**	
	**Odds ratio (95% CI)**	**Adjusted p-value^a, b^**	**Odds ratio (95% CI)**	**Adjusted p-value^a, b^**	**Odds ratio (95% CI)**	**Adjusted p-value^a, b^**
**Diseases**						
HIV	1.01 (0.36–2.88)	0.978	0.46 (0.16–1.30)	0.268	0.56 (0.20–1.60)	0.698
Syphilis	1.28 (0.96–1.72)	0.267	1.62 (1.17–2.26)	**0.026**	1.46 (1.06–2.00)	0.180^**e**^
Gonorrhea	1.94 (1.45–2.59)	**7.9** ^*^10^**−5**^	2.05 (1.45–2.90)	**0.004**	2.13 (1.53–2.95)	**3.6** ^*^10^**−4**^
Genital warts	0.80 (0.59–1.10)	0.380	1.53 (1.08–2.16)	0.064^**e**^	1.68 (1.21–2.33)	**0.030**
Genital herpes	0.88 (0.67–1.17)	0.608	1.38 (1.00–1.90)	0.131	1.02 (0.75–1.39)	0.971
Chlamydia	1.08 (0.78–1.50)	0.769	1.48 (1.04–2.12)	0.107^**e**^	1.37 (0.97–1.93)	0.355
Trichomoniasis	0.88 (0.63–1.25)	0.673	1.50 (1.03–2.18)	0.109^**e**^	1.20 (0.83–1.74)	0.747
Molluscum	1.08 (0.79–1.47)	0.769	1.97 (1.41–2.77)	**0.011**	1.30 (0.94–1.82)	0.462
Scabies and pediculosis	0.88 (0.66–1.16)	0.593	1.70 (1.23–2.35)	**0.011**	0.97 (0.71–1.31)	0.971
Hepatitis B and C	0.95 (0.72–1.25)	0.798	1.40 (1.01–1.94)	0.113^**e**^	0.94 (0.69–1.27)	0.971
**Symptoms**						
Groin swelling	0.93 (0.70–1.22)	0.745	1.64 (1.18–2.27)	**0.023**	1.28 (0.94–1.74)	0.462
Genital ulcers	0.54 (0.38–0.76)	**0.004**	1.90 (1.20–3.01)	**0.034**	1.06 (0.72–1.56)	0.971
Genital itching	0.83 (0.61–1.13)	0.450	1.71 (1.15–2.54)	**0.035**	1.89 (1.30–2.76)	**0.018**
Genital rash	0.88 (0.64–1.20)	0.620	1.53 (1.04–2.27)	0.107^**e**^	1.28 (0.89–1.83)	0.585
Groin pain	0.75 (0.56–0.99)	0.157^**e**^	1.68 (1.20–2.35)	0.054^**e**^	1.03 (0.76–1.40)	0.971
Painful urination	1.17 (0.88–1.55)	0.518	1.19 (0.85–1.65)	0.467	1.19 (0.87–1.63)	0.698
Menstrual issues	1.07 (0.81–1.41)	0.769	1.27 (0.92–1.75)	0.285	1.20 (0.88–1.63)	0.695
Vaginal discharge	0.85 (0.63–1.13)	0.483	1.45 (1.02–2.08)	0.113^**e**^	1.26 (0.90–1.75)	0.573
Urethral discharge	0.88 (0.67–1.17)	0.608	1.55 (1.10–2.17)	**0.020**	1.09 (0.79–1.49)	0.954
Body rash	0.98 (0.74–1.30)	0.932	1.22 (0.88–1.68)	0.396	1.01 (0.74–1.38)	0.986
Fever	0.92 (0.70–1.22)	0.745	1.69 (1.23–2.34)	**0.011**	1.00 (0.73–1.36)	0.996
Frequent diarrhea	1.17 (0.85–1.59)	0.567	1.23 (0.86–1.75)	0.419	0.88 (0.62–1.24)	0.908
Frequent coughing	1.33 (0.95–1.87)	0.267	1.41 (0.96–2.06)	0.182	1.05 (0.72–1.53)	0.971
Frequent Sore throat	1.13 (0.79–1.60)	0.745	1.22 (0.82–1.82)	0.459	1.08 (0.73–1.58)	0.971
Weight loss	0.94 (0.71–1.26)	0.794	1.21 (0.87–1.68)	0.418	0.84 (0.61–1.15)	0.698
No symptoms	1.14 (0.79–1.64)	0.664	1.45 (0.93–2.27)	0.217	1.11 (0.74–1.65)	0.961
**Transmission**						
Sexual intercourse	0.57 (0.19–1.72)	0.549	0.69 (0.21–0.28)	0.724	0.85 (0.26–2.79)	0.971
Skin contact	0.89 (0.67–1.19)	0.640	1.71 (1.24–2.37)	**0.011**	1.27 (0.93–1.73)	0.500
Sharing objects	0.45 (0.34–0.61)	**1.8** ^*^ **10** ^**−6**^	1.43 (1.00–2.05)	0.131	0.97 (0.70–1.35)	0.971
Sharing food^**c**^	0.66 (0.50–0.88)	**0.020**	0.52 (0.37–0.72)	**0.004**	0.92 (0.67–1.24)	0.947
Swimming pools^**c**^	2.42 (1.73–3.40)	**1.8** ^*^ **10** ^**−6**^	0.65 (0.43–0.99)	0.117^**e**^	0.90 (0.62–1.31)	0.947
Blood and injections	1.54 (0.96–2.47)	0.216	1.75 (0.97–3.18)	0.153	1.10 (0.66–1.81)	0.971
Hairdressing	0.82 (0.59–1.13)	0.430	1.98 (1.30–3.02)	**0.011**	1.24 (0.86–1.79)	0.675
Pregnancy and childbirth	1.30 (0.98–1.72)	0.209	1.61 (1.16–2.24)	**0.026**	1.43 (1.05–1.95)	0.180^**e**^
Breastfeeding	1.90 (1.43–2.53)	**7.9** ^*^10^**−5**^	1.59 (1.15–2.20)	**0.030**	1.37 (1.01–1.87)	0.259^**e**^
Mosquito bite^**c**^	0.85 (0.64–1.13)	0.483	0.65 (0.46–0.91)	0.052^**e**^	0.77 (0.56–1.06)	0.462
**Risk factors**						
Multiple partners	1.28 (0.56–3.05)	0.745	1.12 (0.41–3.06)	0.892	1.07 (0.42–2.76)	0.971
Unprotected sex	2.55 (1.67–3.90)	**9** ^*^ **10** ^**−5**^	1.40 (0.88–2.21)	0.285	1.07 (0.71–1.63)	0.971
Substance use	0.76 (0.56–1.03)	0.216	1.30 (0.90–1.86)	0.290	0.73 (0.53–1.01)	0.300
Prostitution	1.28 (0.74–2.21)	0.608	1.42 (0.72–2.78)	0.467	1.40 (0.74–2.64)	0.720
STI co-infection	1.16 (0.68–1.98)	0.745	2.20 (1.03–4.71)	0.113^**e**^	1.20 (0.66–2.20)	0.947
Multiple marriages	0.28 (0.21–0.39)	**3.6** ^*^ **10** ^**−14**^	1.47 (1.02–2.13)	0.113^**e**^	0.91 (0.66–1.27)	0.947
**Prevention**						
Abstinence^**d**^	0.99 (0.75–1.31)	0.976	1.11 (0.80–1.53)	0.724	0.73 (0.54–1.00)	0.259^**e**^
Condoms	2.06 (1.42–3.00)	**0.001**	1.51 (0.98–2.32)	0.153	1.59 (1.05–2.40)	0.187^**e**^
Single partner	0.33 (0.21–0.51)	**3** ^*^ **10** ^**−6**^	0.82 (0.51–1.31)	0.591	0.63 (0.41–0.99)	0.259^**e**^
Routine check-up	0.36 (0.18–0.70)	**0.011**	1.21 (0.55–2.69)	0.768	0.66 (0.34–1.30)	0.675
Vaccines (warts)	0.64 (0.42–0.96)	0.115^**e**^	0.98 (0.61–1.57)	0.953	1.04 (0.66–1.63)	0.971
Vaccines (HIV)^**c**^	1.51 (1.13–2.00)	**0.024**	0.70 (0.51–0.97)	0.107^**e**^	0.93 (0.68–1.26)	0.971
Showering before and after sex^**c**^	1.38 (0.83–2.30)	0.425	0.88 (0.47–1.62)	0.783	1.01 (0.58–1.78)	0.995
Contraceptive pill	1.01 (0.76–1.34)	0.976	0.69 (0.50–0.95)	0.098^**e**^	1.02 (0.74–1.39)	0.975
Circumcision	0.96 (0.70–1.31)	0.854	0.98 (0.68–1.39)	0.933	1.03 (0.73–1.45)	0.971
**Outcome**						
Resolution (HIV)^**c**^	0.91 (0.66–1.26)	0.745	0.67 (0.47–0.96)	0.107^**e**^	0.60 (0.43– 0.85)	0.051^**e**^
Resolution (others)^**c**^	0.76 (0.57–1.02)	0.209	0.78 (0.56–1.09)	0.270	0.95 (0.69–1.30)	0.971
Infertility	1.01 (0.76–1.34)	0.976	1.08 (0.78–1.49)	0.772	1.11 (0.82–1.51)	0.947
Abortion	0.75 (0.56–1.00)	0.173^**e**^	1.30 (0.92–1.83)	0.268	0.98 (0.71–1.34)	0.971
Premature birth	0.76 (0.57–1.00)	0.187	1.10 (0.80–1.52)	0.736	1.46 (1.07–1.98)	0.170^**e**^
Birth defects	0.95 (0.72–1.27)	0.798	1.11 (0.80–1.54)	0.724	1.13 (0.82–1.55)	0.908
Kidney problems	1.30 (0.94–1.81)	0.293	1.89 (1.24–2.87)	**0.023**	1.24 (0.86–1.79)	0.675
Cancer	0.79 (0.59–1.00)	0.275	1.23 (0.88–1.72)	0.396	1.03 (0.75–1.41)	0.971
Death	1.05 (0.79–1.41)	0.798	1.35 (0.95–1.91)	0.212	0.89 (0.65–1.22)	0.908
**Information source**						
School	1.02 (0.77–1.35)	0.932	0.89 (0.65–1.23)	0.669	0.91 (0.67–1.23)	0.947
Healthcare providers	1.23 (0.93–1.63)	0.351	1.63 (1.18–2.26)	**0.023**	1.29 (0.94–1.75)	0.462
Parents	0.81 (0.60–1.10)	0.383	1.59 (1.14–2.23)	**0.035**	0.99 (0.71–1.39)	0.995
Friends	2.45 (1.84–3.27)	**2.1** ^*^ **10** ^**−8**^	1.71 (1.23–2.38)	**0.011**	2.04 (1.49–2.80)	**3.6** ^*^ **10** ^**−4**^
Books	1.26 (0.95–1.67)	0.293	1.28 (0.92–1.79)	0.270	1.71 (1.24–2.36)	**0.018**
TV	1.07 (0.81–1.41)	0.769	1.07 (0.77–1.48)	0.787	1.23 (0.90–1.67)	0.608
The Internet	1.54 (0.87–2.74)	0.333	1.21 (0.63–2.33)	0.736	1.20 (0.64–2.23)	0.947

^a^Chi-square test was utilized to test for association with a 0.05 cut-off point for statistical significance.

^b^Benjamin-Hochberg procedure was utilized to adjust for multiple tested and reduce the false discovery rate to 5% within each family of hypothesis testing (within each individual independent variable).

^**c**^For these questions, “No” was the correct answer.

^**d**^During data collection, abstinence was described as restraining from sexual experience before marriage.

^**e**^These items showed statistically significant associations before adjusting for multiple testing.

**Supplementary Table 3:** Odds ratios and adjusted p-values for attitudes toward sexually transmitted infections, their prevention, and infected individuals among non-medical undergraduates in Baghdad, Iraq.

**Categories**	**Gender**		**Do you know someone who has been diagnosed with an STI?**	
	**Odds ratio (95% CI)**	**Adjusted p-value^a, b^**	**Odds ratio (95% CI)**	**Adjusted p-value^a, b^**
**Sexually transmitted infections**				
Can be effectively prevented	0.85 (0.43–1.70)	0.769	1.01 (0.45–2.27)	0.982
**Public health campaigns**				
Have made you reconsider sex	0.86 (0.60–1.24)	0.620	1.27 (0.82–1.98)	0.459
More campaigns are needed	0.88 (0.42–1.84)	0.798	1.25 (0.50–3.11)	0.768
**Sex education**				
Should be taught in middle/high school	0.85 (0.58–1.26)	0.620	1.44 (0.88–2.35)	0.459
Should be a part of science class	0.62 (0.42–0.90)	0.058^**c**^	0.93 (0.60–1.45)	0.768
**Condoms**				
Can cause infertility	0.70 (0.50–0.99)	0.161^**c**^	1.19 (0.82–1.74)	0.531
Can increase participation in casual sex	0.63 (0.47–0.83)	**0.006**	0.78 (0.56–1.08)	0.268
Can decrease sexual pleasure	3.14 (2.22–4.45)	**1.4** ^*^ **10** ^**−9**^	1.37 (0.95–1.99)	0.212
Can lead to partner mistrust	1.58 (1.19–2.11)	**0.011**	1.07 (0.77–1.48)	0.787
Are not effective when used as the only infection prevention method	0.52 (0.39–0.70)	**9** ^*^ **10** ^**−5**^	0.92 (0.66–1.29)	0.768
**Individuals with STIs**				
Should be socially isolated	1.23 (0.92–1.64)	0.358	1.05 (0.76–1.46)	0.844
Should suffer from violence	1.56 (1.17–2.07)	0.383	1.14 (0.83–1.58)	0.933
Should have fewer jobs	1.22 (0.91–1.65)	**0.011**	0.97 (0.69–1.38)	0.596
Should be stigmatized by doctors	1.29 (0.97–1.70)	0.221	1.05 (0.76–1.45)	0.844
**Categories**	**Previous sexual experience**			
	**Odds ratio (95% CI)**		**Adjusted p-value** ^a, b^	
**Sexually transmitted infections**				
Can be effectively prevented	1.48 (0.64–3.45)		0.788	
**Public health campaigns**				
Have made you reconsider sex	0.97 (0.65–1.45)		0.971	
More campaigns are needed	1.04 (0.46–2.38)		0.975	
**Sex education**				
Should be taught in middle/high school	1.14 (0.73–1.79)		0.947	
Should be a part of science class	0.97 (0.63–1.48)		0.971	
**Condoms**				
Can cause infertility	0.83 (0.57–1.21)		0.747	
Can increase participation in casual sex	0.54 (0.40–0.74)		**0.002**	
Can decrease sexual pleasure	1.50 (1.05–2.15)		0.180^**c**^	
Can lead to partner mistrust	0.74 (0.54–1.00)		0.270	
Are not effective when used as the only infection prevention method	0.79 (0.57–1.08)		0.500	
**Individuals with STIs**				
Should be socially isolated	1.00 (0.73–1.37)		0.996	
Should suffer from violence	0.89 (0.65–1.21)		0.975	
Should have fewer jobs	0.98 (0.71–1.36)		0.908	
Should be stigmatized by doctors	0.93 (0.69–1.27)		0.971	

^a^Chi-square test was utilized to test for association with a 0.05 cut-off point for statistical significance.

^b^Benjamin-Hochberg procedure was utilized to adjust for multiple tested and reduce the false discovery rate to 5% within each family of hypothesis testing (within each individual independent variable).

^c^These items showed statistically significant associations before adjusting for multiple testing.

**Supplementary Table 4:** Odds ratios and adjusted p-values for practices upon suspicion or diagnosis with a sexually transmitted infection among non-medical undergraduates in Baghdad, Iraq.

**Categories**	**Gender**		**Do you know someone who has been diagnosed with an STI?**	
	**Odds ratio (95% CI)**	**Adjusted p-value^a, b^**	**Odds ratio (95% CI)**	**Adjusted p-value^a, b^**
**Suspicion of having an STI due to symptoms or after high-risk behavior**				
Ask your parent	0.94 (0.71–1.25)	0.775	0.99 (0.71–1.37)	0.957
Ask a friend	1.72 (1.29–2.29)	**0.001**	1.10 (0.79–1.53)	0.736
Seek medical advice	1.98 (1.08–3.63)	0.107^**d**^	0.95 (0.51–1.78)	0.933
Search the internet	0.79 (0.50–1.24)	0.533	1.01 (0.59–1.74)	0.969
Ignore this suspicion if no symptoms	1.20 (0.89–1.62)	0.450	0.95 (0.67–1.35)	0.844
**Diagnosis with an STI**				
Follow the doctor's advice	0.84 (0.39–1.82)	0.769	0.73 (0.31–1.69)	0.648
Self-medicate with OTC drugs^**c**^	0.87 (0.60–1.26)	0.664	1.57 (1.05–2.32)	0.102^**d**^
Seek herbal and traditional medicine	1.25 (0.92–1.69)	0.358	1.24 (0.87–1.75)	0.396
Ignore the diagnosis if mild	0.84 (0.57–1.24)	0.608	1.41 (0.93–2.14)	0.227
**Categories**	**Previous sexual experience**			
	**Odds ratio (95% CI)**		**Adjusted p-value** ^a, b^	
**Suspicion of having an STI due to symptoms or after high-risk behavior**				
Ask your parent	0.63 (0.45–0.87)		0.056^**d**^	
Ask a friend	1.25 (0.92–1.71)		0.554	
Seek medical advice	1.30 (0.69–2.47)		0.889	
Search the internet	0.95 (0.57–1.58)		0.971	
Ignore this suspicion if no symptoms	0.91 (0.65–1.27)		0.947	
**Diagnosis with an STI**				
Follow the doctor's advice	0.75 (0.33–1.69)		0.933	
Self-medicate with OTC drugs^**c**^	1.22 (0.83–1.80)		0.747	
Seek herbal and traditional medicine	0.96 (0.68–1.35)		0.971	
Ignore the diagnosis if mild	0.97 (0.64–1.48)		0.971	

^a^Chi-square test was utilized to test for association with a 0.05 cut-off point for statistical significance.

^b^Benjamin-Hochberg procedure was utilized to adjust for multiple tested and reduce the false discovery rate to 5% within each family of hypothesis testing (within each individual independent variable).

^c^OTC, over the counter.

^**d**^These items showed statistically significant associations before adjusting for multiple testing.

These supplementary analyses may not change the scientific conclusions of the study. However, they identify areas where more caution should be employed when discussing certain significant associations. Namely, the higher knowledge of the genital warts' vaccine and the lower institutional stigma among female students, as well as the higher tendency for self-medication with over-the-counter drugs among those who knew someone with a sexually transmitted infection. Future studies should place higher emphasis on investigating these associations in depth, as they cannot be established with certainty based on these adjustments.

The authors apologize for this error and state that this does not change the scientific conclusions of the article in any way. The original article has been updated.
