# Supplementary material for: Assessing knowledge, attitudes, and practices toward sexually transmitted infections among Baghdad undergraduate students for research-guided sexual health education
Source: Front Public Health. 2023 Feb 16;11:1017300. doi: 10.3389/fpubh.2023.1017300 (PMC9980901; doi:10.3389/fpubh.2023.1017300)
Supplement: Supplementary file 1 [file Presentation_1.zip › Appendix A. Research questionnnaire.pdf]

Knowledge, attitude and practices toward sexually transmitted infections among non-medical undergraduates in Baghdad

* Required

This questionnaire belongs to a medical research whose purpose is to study awareness toward sexually transmitted infections among non-medical undergraduate college students from Baghdad. The study was approved by the researchers' institution (University of Baghdad/College of medicine) as well as ministry of health and environment

Participants will be completely anonymous with no personal information that could identify them during the whole process of data collection, data analysis and written the research paper itself

Disclaimer: By clicking on the "submit" button, the participant consent to using their answers for medical research including sharing of raw data (with all participants' answers before analysis) to a data repository

1. Age (in years) *

____

2. Gender *

   Mark only one oval.

   ◯ Male

   ◯ Female

3. Ethnicity *

Mark only one oval.

- ⬭ Arab
- ⬭ Kurd
- ⬭ Other minorities

4. Religion *

Mark only one oval.

- ⬭ Muslim
- ⬭ Believe in another religion
- ⬭ Not religious

5. Residency status *

Mark only one oval.

- ⬭ Urban
- ⬭ Rural

6. Marital experience *

Mark only one oval.

- ⬭ Married/Divorced/Widow
- ⬭ No marital experience

7. Are you currently enrolled as an undergraduate college student? *

Mark only one oval.

- Yes
- No

8. University *

____

9. Field of study *

____

10. Do you know someone who has been diagnosed with a sexually transmitted infection? *

Mark only one oval.

- Yes
- No

11. Do you have a previous sexual experience (of any types) *

Mark only one oval.

- Yes
- No

Knowledge of sexually transmitted infections:

12. How would you rate your knowledge of sexually transmitted infections? *

Mark only one oval.

1	2	3	4	5	6	7	8	9	10
◯	◯	◯	◯	◯	◯	◯	◯	◯	◯

13. Do you recognize the following as sexually transmitted infections? * 10 points

Mark only one oval per row.

	Yes	No
**HIV**	◯	◯
**Syphilis**	◯	◯
**Gonorrhea**	◯	◯
**Genital warts**	◯	◯
**Genital Herpes**	◯	◯
**Chlamydia**	◯	◯
**Trachomonitis**	◯	◯
**Molloscum**	◯	◯
**Scabies and pediculosis**	◯	◯
**Hepatitis B and C**	◯	◯

14. Do you recognize the following as features of sexually transmitted infections ? * 15 points

Mark only one oval per row.

	Yes	No
**Groin swelling**	⬭	⬭
**Genital ulcers**	⬭	⬭
**Genital Itching**	⬭	⬭
**Genital rash**	⬭	⬭
**Groin pain**	⬭	⬭
**Painful urination**	⬭	⬭
**Menstrual problems**	⬭	⬭
**Vaginal discharge**	⬭	⬭
**Urethral discharge**	⬭	⬭
**Body rash**	⬭	⬭
**Fever**	⬭	⬭
**Frequent diarrhea**	⬭	⬭
**Frequent cough**	⬭	⬭
**Frequent sore throat**	⬭	⬭
**Weight loss**	⬭	⬭

15. Someone could have a sexually transmitted infection but shows no symptoms * 1 point

Mark only one oval.

- Yes
- No

16. Do you recognize the following as transmission methods for sexually transmitted infections? * 10 points

Mark only one oval per row.

	Yes	No
**Sexual intercourse**		
**Non-sexual skin contact**		
**Indirectly through objects**		
**Sharing food and drinks**		
**Swimming pools**		
**Blood transfusion and injections**		
**Hairdressing**		
**Pregnancy and childbirth**		
**Breastfeeding**		
**Mosquito bite**		

17. Do you recognize the following as risk factors of acquiring sexual transmitted infections? * 5 points

Mark only one oval per row.

	Yes	No
**Multiple partners**	◯	◯
**Unprotectd sex**	◯	◯
**Substance use**	◯	◯
**Prostitution**	◯	◯
**Having another untreated sexually transmitted infection**	◯	◯

18. Multiple marriages can increase the Transmission of sexually transmitted infections * 1 point

Mark only one oval.

◯ Yes

◯ No

19. Do you think the following are protective acts against sexually transmitted infections? * 8 points

Mark only one oval per row.

	Yes	No
**Abstinence before marriage**	⬭	⬭
**Condoms**	⬭	⬭
**Single partner**	⬭	⬭
**Routine check-ups**	⬭	⬭
**Vaccination for genital warts**	⬭	⬭
**Showering before and after sexual intercourse**	⬭	⬭
**Hormonal contraceptive**	⬭	⬭
**Circumcision**	⬭	⬭

20. Do you recognize the following as complications of sexually transmitted infections? * 7 points

Mark only one oval per row.

	Yes	No
**Infertility**	⬭	⬭
**Abortion**	⬭	⬭
**Premature birth**	⬭	⬭
**Birth defects**	⬭	⬭
**Kidney problems**	⬭	⬭
**Cancer**	⬭	⬭
**Death**	⬭	⬭

21. Do you Agree with the following statements * 3 points

Mark only one oval per row.

	Yes	No
**Individuals with HIV can reach permanent resolution**	⬭	⬭
**All sexually transmitted infections other than HIV can reach permanent resolution**	⬭	⬭
**There is a vaccine against HIV**	⬭	⬭

22. What is your source of information? *

Mark only one oval per row.

	Yes	No
**School**	⬭	⬭
**Healthcare workers**	⬭	⬭
**Parents**	⬭	⬭
**Friends**	⬭	⬭
**Books**	⬭	⬭
**TV**	⬭	⬭
**The internet**	⬭	⬭

Attitudes toward sexually transmitted infections:

23. Do you agree with the following statements? * 0 points

Mark only one oval per row.

	Yes	No
**sexually transmitted infections can be effectively prevented**		
**Public health campaigns have made you reconsider your sexual practices**		
**More public health campaigns are needed**		
**Sex education should be taught in middle/high school**		
**If taught, sex education should be a part of science class?**		

24. What do you think is the most important barrier against sex education? *

Mark only one oval.

- Sensitivity of the subject
- Religious barriers
- Traditional barriers

25. The follows are concerns I have about condoms usage *

Mark only one oval per row.

	Yes	No
**Condoms can cause infertility**		
**Condoms can increase participation in causal sex**		
**Condoms can decrease the pleasure of sexual activity**		
**Condoms could lead to partner distrust**		
**Condoms are not effective if used as the only infection prevention method**		

26. Do you agree with the following statements? *

Mark only one oval per row.

	Yes	No
**Individuals with sexually transmitted infections should be socially isolated**	◯	◯
**Individuals with sexually transmitted infections should have less job opportunities**	◯	◯
**Individuals with sexually transmitted infections are should suffer from interpersonal violence**	◯	◯
**Individuals with sexually transmitted infections should be stigmatized by healthcare workers**	◯	◯

27. if you had a suspicion of having a sexually transmitted infection due to symptoms * or after high risk behavior, would you:

Mark only one oval per row.

	Yes	No
**Consult your parents**	◯	◯
**Consult a Friend**	◯	◯
**Directly seek medical advice**	◯	◯
**Search the internet**	◯	◯
**Ignore the suspicion if no symptoms**	◯	◯

28. If you get diagnosed with a sexually transmitted infection would you: *

Mark only one oval per row.

	Yes	No
**Follow the doctor's advice**	◯	◯
**Self-medicate with over the counter drugs**	◯	◯
**Seek herbal and traditional medicine**	◯	◯
**Ignore the diagnosis if mild**	◯	◯

Disclaimer: By clicking on the "submit" button, the participant consent to using their answers for medical research including sharing of raw data (with all participants' answers before analysis) to a data repository

This content is neither created nor endorsed by Google.

Google Forms
