# Supplementary material for: Assessing knowledge, attitudes, and practices toward sexually transmitted infections among Baghdad undergraduate students for research-guided sexual health education
Source: Front Public Health. 2023 Feb 16;11:1017300. doi: 10.3389/fpubh.2023.1017300 (PMC9980901; doi:10.3389/fpubh.2023.1017300)
Supplement: Supplementary file 1 [file Presentation_1.zip › Appendix B. Knowledge supplementary materials.docx]

Appendix B

**Table B1** | Knowledge score across three different demographic factors

| **Variables** | **Groups** | **Mean score** | **Mean difference** | **Equal variances assumed ^a^** | **Equal variance not assumed ^a^** |
| --- | --- | --- | --- | --- | --- |
| Gender | Male | 34.822 (±6.8) | 0.450 | t = - 0.952  p-value = .341 | t = - 0.944  p-value = .345 |
|  | Female | 35.272 (±6.5) |  |  |  |
| Do you know someone who has been diagnosed with an STD? | Yes | 37.174 (±6.7) | 2.730 | t = 5.076  **p-value = 4.7*10^^-7^** | t = 4.970  **p-value = 1*10^^-6^** |
|  | No | 34.444 (±6.4) |  |  |  |
| Previous sexual experience | Yes | 35.769 (±7.0) | 0.935 | t = 1.802  p-value = .072 | t = 1.734  p-value = .084 |
|  | No | 34.834 (±6.4) |  |  |  |

**^a^** 0.05 is the cutoff point, significant results are indicated with a **bold** text

**Table B2** | Common misconceptions

| **Categories with >50% incorrect responses** | **No. of individuals (%)** |
| --- | --- |
| **STDs**  Trichomoniasis  Chlamydia  Warts  Molluscum  Syphilis  Scabies  Genital herpes  Hepatitis B and C | 652 (79.2)  622 (75.5)  596 (72.4)  590 (71.7) 543 (66.0)  450 (54.7) 426 (51.8)  420 (51.0) |
| **Symptoms**  Frequent sore throat  Frequent cough  Frequent diarrhea  Weight loss  Body rash  Fever | 666 (80.9)  653 (79.3)  600 (72.9)  514 (62.5)  486 (59.1) 466 (56.6) |
| **Transmission**  Swimming pools  Non-sexual skin contact  Mosquitos bites  Breastfeeding | 645 (78.4) 500 (60.8) 484 (58.8)  469 (57.0) |
| **Protective factors**  Showering before and after sex  Abstinence before marriage | 758 (92.1)  438 (53.2) |
| **Outcome**  All STDs other than HIV can reach a resolution  Premature birth | 499 (60.6)  420 (51.0) |
