# Supplementary material for: Assessing knowledge, attitudes, and practices toward sexually transmitted infections among Baghdad undergraduate students for research-guided sexual health education
Source: Front Public Health. 2023 Feb 16;11:1017300. doi: 10.3389/fpubh.2023.1017300 (PMC9980901; doi:10.3389/fpubh.2023.1017300)
Supplement: Supplementary file 1 [file Presentation_1.zip › Appendix C. Attitudes supplementary materials.docx]

Appendix C

# Table C1 | Association between acceptance toward the implementation of sex education programs and perceived barriers

| **Barriers against sex**  **education** | **Sex education should be taught in**  **middle/high school** | | | **p-value ^a^** |
| --- | --- | --- | --- | --- |
|  | **Yes** | **No** | **Total** |  |
| Sensitivity of the subject | 173 | 48 | 221 | **9.5*10^^-7^** |
|  | 24.57% | 40.34% | 26.85% |  |
| Traditional Barriers | 456 | 47 | 503 |  |
|  | 64.77% | 39.5% | 61.11% |  |
| Religious barriers | 75 | 24 | 99 |  |
|  | 10.65% | 20.17% | 12.09% |  |

**^a^** Chi-square for association with a cutoff point of 0.05 for p-value and significant results indicated with a **bold** text

# Table C2 | Association between acceptance toward the implementation of sex education programs and knowledge score

| **Variables** | **Sex education should be taught in school** | **Mean score** | **Mean difference** | **Equal variances assumed^a^** | **Equal variance not assumed^a^** |
| --- | --- | --- | --- | --- | --- |
| Knowledge score | Yes | 35.424  (±6.6) | 2.307 | t = 3.519  **p-value = 4.5*10^^-4^** | t = 3.517  **p-value = 0.001** |
|  | No | 33.117  (±6.6) |  |  |  |

**^a^** 0.05 is the cutoff point, significant results are indicated with a **bold** text

# Table C3 | Association between social stigmatization and misconceptions regarding transmission during social activities

| **Social activity** | **Groups** | **Individuals with sexually transmitted diseases should be socially isolated** | | | **p-value ^b^** |
| --- | --- | --- | --- | --- | --- |
|  |  | **Yes**  **499 (%)^a^** | **No**  **324 (%)^a^** | **Total**  **823 (%)^a^** |  |
| Sharing food and drinks | Yes | 244 (67.4) | 118 (32.6) | 362 (100.0) | **4.2*10^^-4^** |
|  | No | 255 (55.3) | 206 (44.7) | 461 (100.0) |  |
| Swimming pool | Yes | 404 (62.6) | 241 (37.4) | 645 (100.0) | **.025** |
|  | No | 95 (53.4) | 83 (46.6) | 178 (100.0) |  |

**^a^** Row percent was used

**^b^** 0.05 is the cutoff point, significant results are indicated with a **bold** text
