# Supplementary material for: Assessing knowledge, attitudes, and practices toward sexually transmitted infections among Baghdad undergraduate students for research-guided sexual health education
Source: Front Public Health. 2023 Feb 16;11:1017300. doi: 10.3389/fpubh.2023.1017300 (PMC9980901; doi:10.3389/fpubh.2023.1017300)
Supplement: Supplementary file 1 [file Presentation_1.zip › Appendix D. Practices supplementary materials.docx]

Appendix D

**Table D1** | Characteristics of Non-medical undergraduates with previous sexual experience

| **Variables** | **Previous sexual experience** | | | **Equal variances assumed ^a^** | **Equal variance not assumed ^a^** |
| --- | --- | --- | --- | --- | --- |
|  | **Yes** | **No** | **Mean difference** |  |  |
| Age (years) | 23.74  (±4.7) | 21.69  (±2.9) | 2.050 | t = 7.399  **p-value = 3.4*10^^-13^** | t = 6.033  **p-value = 4.8*10^^-9^** |
| **Variables** | **Comparison Groups** | **Previous sexual experience** | | **Chi-square** | **p-value ^a^** |
|  |  | **Yes** | **No** |  |  |
| Gender **^b^** | Male | 137  41.3% | 195  58.7% | 53.238 | **2.9*10^^-13^** |
|  | Female | 89  18.1% | 402  81.9% |  |  |
| Do you know someone who has been diagnosed with STD? **^b^** | Yes | 79  40.5% | 116  59.5% | 21.856 | **3.0*10^^-6^** |
|  | No | 147  23.4% | 481  76.6% |  |  |

**^a^** 0.05 is the cutoff point, significant results are indicated with a **bold** text

**^b^** Row percent was used for these variables
