# Supplementary material for: Assessing knowledge, attitudes, and practices toward sexually transmitted infections among Baghdad undergraduate students for research-guided sexual health education
Source: Front Public Health. 2023 Feb 16;11:1017300. doi: 10.3389/fpubh.2023.1017300 (PMC9980901; doi:10.3389/fpubh.2023.1017300)
Supplement: Supplementary file 2 [file Table_1.docx]

**Supplementary Table 1**. Effect size indices for independent samples T-test (N = 823).

| **Scale** | **Categorical variable** | **Groups** | **Mean (SD)** | **Mean difference** | **Levene’s**  **Test** | **T-test** | **Effect size** |
| --- | --- | --- | --- | --- | --- | --- | --- |
| Knowledge score | Gender | Male | 34.82 (±6.8) | 0.450 | F = 1.359  p = 0.244 | t = -0.952**^a^**  p = 0.341 | -0.068**^c^** |
|  |  | Female | 35.27 (±6.5) |  |  |  |  |
|  | Do you know someone with an STD? | Yes | 37.17 (±6.7) | 2.730 | F = 0.121  p = 0.728 | t = 5.076**^a^**  p = **4.8*10^-7^** | 0.416**^c^** |
|  |  | No | 34.44 (±6.4) |  |  |  |  |
|  | Previous sexual experience | Yes | 35.77 (±7.0) | 0.935 | F = 2.317  p = 0.128 | t = 1.802**^a^**  p = 0.072 | 0.141**^c^** |
|  |  | No | 34.83 (±6.4) |  |  |  |  |
|  | Sex education should be taught in school | Yes | 35.42 (±6.6) | 2.307 | F = 0.249  p = 0.618 | t = 3.519**^a^**  p = **4.5*10^-4^** | 0.349**^c^** |
|  |  | No | 33.11 (±6.6) |  |  |  |  |
| Age (Years) | Previous sexual experience | Yes | 23.74 (±4.7) | 2.050 | F = 34.015  p = **7.9*10^-9^** | t = 6.033**^b^**  p = **4.8*10^-9^** | 0.525**^d^** |
|  |  | No | 21.69 (±2.9) |  |  |  |  |

**^a^ Student’s independent samples T-test** was used to test for association with 0.05 as a cut-off point for statistical significance.
**^b^ Welch’s independent samples T-test** was used to test for association with 0.05 as a cut-off point for statistical significance.
**^c^ Cohen’s D** was used to assess effect size with ≥0.2 for small, ≥0.5 for moderate, and ≥0.8 for large effect sizes.
**^d^** **The mean difference scaled by the square root of the average variance** was used to assess effect size with ≥0.2 for small, ≥0.5 for moderate, and ≥0.8 for large effect sizes.

**Supplementary Table 2**. Odds ratios and Adjusted p-values for the knowledge about sexually transmitted infections among non-medical undergraduates in Baghdad, Iraq**.**

| **Category** | **Gender** | | **Do you know someone who has been diagnosed with an STI?** | | | **Previous sexual experience** | | |
| --- | --- | --- | --- | --- | --- | --- | --- | --- |
|  | **Odds ratio**  **(95% CI)** | **Adjusted**  **p-value^a, b^** | **Odds ratio**  **(95% CI)** | | **Adjusted**  **p-value^a, b^** | **Odds ratio**  **(95% CI)** | | **Adjusted**  **p-value^a, b^** |
| **Diseases** |  |  |  |  |  |  |  |  |
| HIV | 1.01 (0.36 – 2.88) | 0.978 | 0.46 (0.16 – 1.30) | | 0.268 | 0.56 (0.20 – 1.60) | | 0.698 |
| Syphilis | 1.28 (0.96 – 1.72) | 0.267 | 1.62 (1.17 – 2.26) | | **0.026** | 1.46 (1.06 – 2.00) | | 0.180 **^e^** |
| Gonorrhea | 1.94 (1.45 – 2.59) | **7.9*10^-5^** | 2.05 (1.45 – 2.90) | | **0.004** | 2.13 (1.53 – 2.95) | | **3.6*10^-4^** |
| Genital warts | 0.80 (0.59 – 1.10) | 0.380 | 1.53 (1.08 – 2.16) | | 0.064 **^e^** | 1.68 (1.21 – 2.33) | | **0.030** |
| Genital herpes | 0.88 (0.67 – 1.17) | 0.608 | 1.38 (1.00 – 1.90) | | 0.131 | 1.02 (0.75 – 1.39) | | 0.971 |
| Chlamydia | 1.08 (0.78 – 1.50) | 0.769 | 1.48 (1.04 – 2.12) | | 0.107 **^e^** | 1.37 (0.97 – 1.93) | | 0.355 |
| Trichomoniasis | 0.88 (0.63 – 1.25) | 0.673 | 1.50 (1.03 – 2.18) | | 0.109 **^e^** | 1.20 (0.83 – 1.74) | | 0.747 |
| Molluscum | 1.08 (0.79 – 1.47) | 0.769 | 1.97 (1.41 – 2.77) | | **0.011** | 1.30 (0.94 – 1.82) | | 0.462 |
| Scabies and pediculosis | 0.88 (0.66 – 1.16) | 0.593 | 1.70 (1.23 – 2.35) | | **0.011** | 0.97 (0.71 – 1.31) | | 0.971 |
| Hepatitis B and C | 0.95 (0.72 – 1.25) | 0.798 | 1.40 (1.01 – 1.94) | | 0.113 **^e^** | 0.94 (0.69 – 1.27) | | 0.971 |
| **Symptoms** |  |  |  | |  |  | |  |
| Groin swelling | 0.93 (0.70 – 1.22) | 0.745 | 1.64 (1.18 – 2.27) | | **0.023** | 1.28 (0.94 – 1.74) | | 0.462 |
| Genital ulcers | 0.54 (0.38 – 0.76) | **0.004** | 1.90 (1.20 – 3.01) | | **0.034** | 1.06 (0.72 – 1.56) | | 0.971 |
| Genital itching | 0.83 (0.61 – 1.13) | 0.450 | 1.71 (1.15 – 2.54) | | **0.035** | 1.89 (1.30 – 2.76) | | **0.018** |
| Genital rash | 0.88 (0.64 – 1.20) | 0.620 | 1.53 (1.04 – 2.27) | | 0.107 **^e^** | 1.28 (0.89 – 1.83) | | 0.585 |
| Groin pain | 0.75 (0.56 – 0.99) | 0.157 **^e^** | 1.68 (1.20 – 2.35) | | 0.054 **^e^** | 1.03 (0.76 – 1.40) | | 0.971 |
| Painful urination | 1.17 (0.88 – 1.55) | 0.518 | 1.19 (0.85 – 1.65) | | 0.467 | 1.19 (0.87 – 1.63) | | 0.698 |
| Menstrual issues | 1.07 (0.81 – 1.41) | 0.769 | 1.27 (0.92 – 1.75) | | 0.285 | 1.20 (0.88 – 1.63) | | 0.695 |
| Vaginal discharge | 0.85 (0.63 – 1.13) | 0.483 | 1.45 (1.02 – 2.08) | | 0.113 **^e^** | 1.26 (0.90 – 1.75) | | 0.573 |
| Urethral discharge | 0.88 (0.67 – 1.17) | 0.608 | 1.55 (1.10 – 2.17) | | **0.020** | 1.09 (0.79 – 1.49) | | 0.954 |
| Body rash | 0.98 (0.74 – 1.30) | 0.932 | 1.22 (0.88 – 1.68) | | 0.396 | 1.01 (0.74 – 1.38) | | 0.986 |
| Fever | 0.92 (0.70 – 1.22) | 0.745 | 1.69 (1.23 – 2.34) | | **0.011** | 1.00 (0.73 – 1.36) | | 0.996 |
| Frequent diarrhea | 1.17 (0.85 – 1.59) | 0.567 | 1.23 (0.86 – 1.75) | | 0.419 | 0.88 (0.62 – 1.24) | | 0.908 |
| Frequent coughing | 1.33 (0.95 – 1.87) | 0.267 | 1.41 (0.96 – 2.06) | | 0.182 | 1.05 (0.72 – 1.53) | | 0.971 |
| Frequent Sore throat | 1.13 (0.79 – 1.60) | 0.745 | 1.22 (0.82 – 1.82) | | 0.459 | 1.08 (0.73 – 1.58) | | 0.971 |
| Weight loss | 0.94 (0.71 – 1.26) | 0.794 | 1.21 (0.87 – 1.68) | | 0.418 | 0.84 (0.61 – 1.15) | | 0.698 |
| No symptoms | 1.14 (0.79 – 1.64) | 0.664 | 1.45 (0.93 – 2.27) | | 0.217 | 1.11 (0.74 – 1.65) | | 0.961 |
| **Transmission** |  |  |  | |  |  | |  |
| Sexual intercourse | 0.57 (0.19 – 1.72) | 0.549 | 0.69 (0.21 – 0.28) | | 0.724 | 0.85 (0.26 – 2.79) | | 0.971 |
| Skin contact | 0.89 (0.67 – 1.19) | 0.640 | 1.71 (1.24 – 2.37) | | **0.011** | 1.27 (0.93 – 1.73) | | 0.500 |
| Sharing objects | 0.45 (0.34 – 0.61) | **1.8*10^-6^** | 1.43 (1.00 – 2.05) | | 0.131 | 0.97 (0.70 – 1.35) | | 0.971 |
| Sharing food**^c^** | 0.66 (0.50 – 0.88) | **0.020** | 0.52 (0.37 – 0.72) | | **0.004** | 0.92 (0.67 – 1.24) | | 0.947 |
| Swimming pools**^c^** | 2.42 (1.73 – 3.40) | **1.8*10^-6^** | 0.65 (0.43 – 0.99) | | 0.117 **^e^** | 0.90 (0.62 – 1.31) | | 0.947 |
| Blood and injections | 1.54 (0.96 – 2.47) | 0.216 | 1.75 (0.97 – 3.18) | | 0.153 | 1.10 (0.66 – 1.81) | | 0.971 |
| Hairdressing | 0.82 (0.59 – 1.13) | 0.430 | 1.98 (1.30 – 3.02) | | **0.011** | 1.24 (0.86 – 1.79) | | 0.675 |
| Pregnancy and childbirth | 1.30 (0.98 – 1.72) | 0.209 | 1.61 (1.16 – 2.24) | | **0.026** | 1.43 (1.05 – 1.95) | | 0.180 **^e^** |
| Breastfeeding | 1.90 (1.43 – 2.53) | **7.9*10^-5^** | 1.59 (1.15 – 2.20) | | **0.030** | 1.37 (1.01 – 1.87) | | 0.259 **^e^** |
| Mosquito bite**^c^** | 0.85 (0.64 – 1.13) | 0.483 | 0.65 (0.46 – 0.91) | | 0.052 **^e^** | 0.77 (0.56 – 1.06) | | 0.462 |

**Supplementary Table 2 (continued)**

| **Category** | **Gender** | | **Do you know someone who has been diagnosed with an STI?** | | **Previous sexual experience** | |
| --- | --- | --- | --- | --- | --- | --- |
|  | **Odds ratio**  **(95% CI)** | **Adjusted**  **p-value^a, b^** | **Odds ratio**  **(95% CI)** | **Adjusted**  **p-value^a, b^** | **Odds ratio**  **(95% CI)** | **Adjusted**  **p-value^a, b^** |
| **Risk factors** |  |  |  |  |  |  |
| Multiple partners | 1.28 (0.56 – 3.05) | 0.745 | 1.12 (0.41 – 3.06) | 0.892 | 1.07 (0.42 – 2.76) | 0.971 |
| Unprotected sex | 2.55 (1.67 – 3.90) | **9*10^-5^** | 1.40 (0.88 – 2.21) | 0.285 | 1.07 (0.71 – 1.63) | 0.971 |
| Substance use | 0.76 (0.56 – 1.03) | 0.216 | 1.30 (0.90 – 1.86) | 0.290 | 0.73 (0.53 – 1.01) | 0.300 |
| Prostitution | 1.28 (0.74 – 2.21) | 0.608 | 1.42 (0.72 – 2.78) | 0.467 | 1.40 (0.74 – 2.64) | 0.720 |
| STI co-infection | 1.16 (0.68 – 1.98) | 0.745 | 2.20 (1.03 – 4.71) | 0.113 **^e^** | 1.20 (0.66 – 2.20) | 0.947 |
| Multiple marriages | 0.28 (0.21 – 0.39) | **3.6*10^-14^** | 1.47 (1.02 – 2.13) | 0.113 **^e^** | 0.91 (0.66 – 1.27) | 0.947 |
| **Prevention** |  |  |  |  |  |  |
| Abstinence**^d^** | 0.99 (0.75 – 1.31) | 0.976 | 1.11 (0.80 – 1.53) | 0.724 | 0.73 (0.54 – 1.00) | 0.259 **^e^** |
| Condoms | 2.06 (1.42 – 3.00) | **0.001** | 1.51 (0.98 – 2.32) | 0.153 | 1.59 (1.05 – 2.40) | 0.187 **^e^** |
| Single partner | 0.33 (0.21 – 0.51) | **3*10^-6^** | 0.82 (0.51 – 1.31) | 0.591 | 0.63 (0.41 – 0.99) | 0.259 **^e^** |
| Routine check-up | 0.36 (0.18 – 0.70) | **0.011** | 1.21 (0.55 – 2.69) | 0.768 | 0.66 (0.34 – 1.30) | 0.675 |
| Vaccines (warts) | 0.64 (0.42 – 0.96) | 0.115 **^e^** | 0.98 (0.61 – 1.57) | 0.953 | 1.04 (0.66 – 1.63) | 0.971 |
| Vaccines (HIV)**^c^** | 1.51 (1.13 – 2.00) | **0.024** | 0.70 (0.51 – 0.97) | 0.107 **^e^** | 0.93 (0.68 – 1.26) | 0.971 |
| Showering before  and after sex**^c^** | 1.38 (0.83 – 2.30) | 0.425 | 0.88 (0.47 – 1.62) | 0.783 | 1.01 (0.58 – 1.78) | 0.995 |
| Contraceptive pill | 1.01 (0.76 – 1.34) | 0.976 | 0.69 (0.50 – 0.95) | 0.098 **^e^** | 1.02 (0.74 – 1.39) | 0.975 |
| Circumcision | 0.96 (0.70 – 1.31) | 0.854 | 0.98 (0.68 – 1.39) | 0.933 | 1.03 (0.73 – 1.45) | 0.971 |
| **Outcome** |  |  |  |  |  |  |
| Resolution (HIV)**^c^** | 0.91 (0.66 – 1.26) | 0.745 | 0.67 (0.47 – 0.96) | 0.107 **^e^** | 0.60 (0.43 – 0.85) | 0.051 **^e^** |
| Resolution (others)**^c^** | 0.76 (0.57 – 1.02) | 0.209 | 0.78 (0.56 – 1.09) | 0.270 | 0.95 (0.69 – 1.30) | 0.971 |
| Infertility | 1.01 (0.76 – 1.34) | 0.976 | 1.08 (0.78 – 1.49) | 0.772 | 1.11 (0.82 – 1.51) | 0.947 |
| Abortion | 0.75 (0.56 – 1.00) | 0.173 **^e^** | 1.30 (0.92 – 1.83) | 0.268 | 0.98 (0.71 – 1.34) | 0.971 |
| Premature birth | 0.76 (0.57 – 1.00) | 0.187 | 1.10 (0.80 – 1.52) | 0.736 | 1.46 (1.07 – 1.98) | 0.170 **^e^** |
| Birth defects | 0.95 (0.72 – 1.27) | 0.798 | 1.11 (0.80 – 1.54) | 0.724 | 1.13 (0.82 – 1.55) | 0.908 |
| Kidney problems | 1.30 (0.94 – 1.81) | 0.293 | 1.89 (1.24 – 2.87) | **0.023** | 1.24 (0.86 – 1.79) | 0.675 |
| Cancer | 0.79 (0.59 – 1.00) | 0.275 | 1.23 (0.88 – 1.72) | 0.396 | 1.03 (0.75 – 1.41) | 0.971 |
| Death | 1.05 (0.79 – 1.41) | 0.798 | 1.35 (0.95 – 1.91) | 0.212 | 0.89 (0.65 – 1.22) | 0.908 |
| **Information source** |  |  |  |  |  |  |
| School | 1.02 (0.77 – 1.35) | 0.932 | 0.89 (0.65 – 1.23) | 0.669 | 0.91 (0.67 – 1.23) | 0.947 |
| Healthcare providers | 1.23 (0.93 – 1.63) | 0.351 | 1.63 (1.18 – 2.26) | **0.023** | 1.29 (0.94 – 1.75) | 0.462 |
| Parents | 0.81 (0.60 – 1.10) | 0.383 | 1.59 (1.14 – 2.23) | **0.035** | 0.99 (0.71 – 1.39) | 0.995 |
| Friends | 2.45 (1.84 – 3.27) | **2.1*10^-8^** | 1.71 (1.23 – 2.38) | **0.011** | 2.04 (1.49 – 2.80) | **3.6*10^-4^** |
| Books | 1.26 (0.95 – 1.67) | 0.293 | 1.28 (0.92 – 1.79) | 0.270 | 1.71 (1.24 – 2.36) | **0.018** |
| TV | 1.07 (0.81 – 1.41) | 0.769 | 1.07 (0.77 – 1.48) | 0.787 | 1.23 (0.90 – 1.67) | 0.608 |
| The Internet | 1.54 (0.87 – 2.74) | 0.333 | 1.21 (0.63 – 2.33) | 0.736 | 1.20 (0.64 – 2.23) | 0.947 |

**^a^ Chi-square test** was utilized to test for association with a 0.05 cut-off point for statistical significance.
**^b^ Benjamin-Hochberg procedure** was utilized to adjust for multiple tested and reduce the false discovery rate to 5% within each family of hypothesis testing (within each individual independent variable).
**^c^** For these questions, “No” was the correct answer.
**^d^** During data collection, abstinence was described as restraining from sexual experience before marriage.
**^e^** These items showed statistically significant associations before adjusting for multiple testing

**Supplementary Table 3**. Odds ratios and Adjusted p-values for attitudes toward sexually transmitted infections, their prevention, and infected individuals among non-medical undergraduates in Baghdad, Iraq.

| **Categories** | **Gender** | | **Do you know someone who has been diagnosed with an STI?** | |
| --- | --- | --- | --- | --- |
|  | **Odds ratio**  **(95% CI)** | **Adjusted**  **p-value^a, b^** | **Odds ratio**  **(95% CI)** | **Adjusted**  **p-value^a, b^** |
| **Sexually transmitted infections** |  |  |  |  |
| can be effectively prevented | 0.85 (0.43 – 1.70) | 0.769 | 1.01 (0.45 – 2.27) | 0.982 |
| **Public health campaigns** |  |  |  |  |
| Have made you reconsider sex | 0.86 (0.60 – 1.24) | 0.620 | 1.27 (0.82 – 1.98) | 0.459 |
| More campaigns are needed | 0.88 (0.42 – 1.84) | 0.798 | 1.25 (0.50 – 3.11) | 0.768 |
| **Sex education** |  |  |  |  |
| Should be taught in middle/high school | 0.85 (0.58 – 1.26) | 0.620 | 1.44 (0.88 – 2.35) | 0.459 |
| Should be a part of science class | 0.62 (0.42 – 0.90) | 0.058 **^c^** | 0.93 (0.60 – 1.45) | 0.768 |
| **Condoms** |  |  |  |  |
| Can cause infertility | 0.70 (0.50 – 0.99) | 0.161 **^c^** | 1.19 (0.82 – 1.74) | 0.531 |
| Can increase participation in casual sex | 0.63 (0.47 – 0.83) | **0.006** | 0.78 (0.56 – 1.08) | 0.268 |
| Can decrease sexual pleasure | 3.14 (2.22 – 4.45) | **1.4*10^-9^** | 1.37 (0.95 – 1.99) | 0.212 |
| Can lead to partner mistrust | 1.58 (1.19 – 2.11) | **0.011** | 1.07 (0.77 – 1.48) | 0.787 |
| Are not effective when used as the only  infection prevention method | 0.52 (0.39 – 0.70) | **9*10^-5^** | 0.92 (0.66 – 1.29) | 0.768 |
| **Individuals with STIs** |  |  |  |  |
| Should be socially isolated | 1.23 (0.92 – 1.64) | 0.358 | 1.05 (0.76 – 1.46) | 0.844 |
| Should suffer from violence | 1.56 (1.17 – 2.07) | 0.383 | 1.14 (0.83 – 1.58) | 0.933 |
| Should have fewer jobs | 1.22 (0.91 – 1.65) | **0.011** | 0.97 (0.69 – 1.38) | 0.596 |
| Should be stigmatized by doctors | 1.29 (0.97 – 1.70) | 0.221 | 1.05 (0.76 – 1.45) | 0.844 |

**Supplementary Table 3 (continued)**

| **Categories** | **Previous sexual experience** | |
| --- | --- | --- |
|  | **Odds ratio**  **(95% CI)** | **Adjusted**  **p-value^a, b^** |
| **Sexually transmitted infections** |  |  |
| can be effectively prevented | 1.48 (0.64 – 3.45) | 0.788 |
| **Public health campaigns** |  |  |
| Have made you reconsider sex | 0.97 (0.65 – 1.45) | 0.971 |
| More campaigns are needed | 1.04 (0.46 – 2.38) | 0.975 |
| **Sex education** |  |  |
| Should be taught in middle/high school | 1.14 (0.73 – 1.79) | 0.947 |
| Should be a part of science class | 0.97 (0.63 – 1.48) | 0.971 |
| **Condoms** |  |  |
| Can cause infertility | 0.83 (0.57 – 1.21) | 0.747 |
| Can increase participation in casual sex | 0.54 (0.40 – 0.74) | **0.002** |
| Can decrease sexual pleasure | 1.50 (1.05 – 2.15) | 0.180 **^c^** |
| Can lead to partner mistrust | 0.74 (0.54 – 1.00) | 0.270 |
| Are not effective when used as the only  infection prevention method | 0.79 (0.57 – 1.08) | 0.500 |
| **Individuals with STIs** |  |  |
| Should be socially isolated | 1.00 (0.73 – 1.37) | 0.996 |
| Should suffer from violence | 0.89 (0.65 – 1.21) | 0.975 |
| Should have fewer jobs | 0.98 (0.71 – 1.36) | 0.908 |
| Should be stigmatized by doctors | 0.93 (0.69 – 1.27) | 0.971 |

**^a^ Chi-square test** was utilized to test for association with a 0.05 cut-off point for statistical significance.
**^b^ Benjamin-Hochberg procedure** was utilized to adjust for multiple tested and reduce the false discovery rate to 5% within each family of hypothesis testing (within each individual independent variable).
**^c^** These items showed statistically significant associations before adjusting for multiple testing.

**Supplementary Table 4**. Odds ratios and Adjusted p-values for practices upon suspicion or diagnosis with a sexually transmitted infection among non-medical undergraduates in Baghdad, Iraq.

| **Categories** | **Gender** | | | | | **Do you know someone who has been diagnosed with an STI?** | | | |
| --- | --- | --- | --- | --- | --- | --- | --- | --- | --- |
|  | **Odds ratio**  **(95% CI)** | | **Adjusted**  **p-value^a, b^** | | | **Odds ratio**  **(95% CI)** | | | **Adjusted**  **p-value^a, b^** |
| **Suspicion of having an STI due to symptoms or after high-risk behavior** |  |  |  | | |  | |  |  |
| ask your parent | 0.94 (0.71 – 1.25) | | 0.775 | | | 0.99 (0.71 – 1.37) | | | 0.957 |
| ask a friend | 1.72 (1.29 – 2.29) | | **0.001** | | | 1.10 (0.79 – 1.53) | | | 0.736 |
| Seek medical advice | 1.98 (1.08 – 3.63) | | 0.107 **^d^** | | | 0.95 (0.51 – 1.78) | | | 0.933 |
| Search the internet | 0.79 (0.50 – 1.24) | | 0.533 | | | 1.01 (0.59 – 1.74) | | | 0.969 |
| Ignore this suspicion if no symptoms | 1.20 (0.89 – 1.62) | | 0.450 | | | 0.95 (0.67 – 1.35) | | | 0.844 |
| **Diagnosis with an STI** |  | |  | | |  | | |  |
| Follow the doctor’s advice | 0.84 (0.39 – 1.82) | | 0.769 | | | 0.73 (0.31 – 1.69) | | | 0.648 |
| Self-medicate with OTC drugs**^c^** | 0.87 (0.60 – 1.26) | | 0.664 | | | 1.57 (1.05 – 2.32) | | | 0.102 **^d^** |
| Seek herbal and traditional medicine | 1.25 (0.92 – 1.69) | | 0.358 | | | 1.24 (0.87 – 1.75) | | | 0.396 |
| Ignore the diagnosis if mild | 0.84 (0.57 – 1.24) | | 0.608 | | | 1.41 (0.93 – 2.14) | | | 0.227 |
| **Categories** | **Previous sexual experience** | | | | | | | | |
|  | **Odds ratio**  **(95% CI)** | | | | **Adjusted**  **p-value^a, b^** | | | | |
| **Suspicion of having an STI due to symptoms or after high-risk behavior** |  | | |  | | |  | | |
| ask your parent | 0.63 (0.45 – 0.87) | | | | 0.056 **^d^** | | | | |
| ask a friend | 1.25 (0.92 – 1.71) | | | | 0.554 | | | | |
| Seek medical advice | 1.30 (0.69 – 2.47) | | | | 0.889 | | | | |
| Search the internet | 0.95 (0.57 – 1.58) | | | | 0.971 | | | | |
| Ignore this suspicion if no symptoms | 0.91 (0.65 – 1.27) | | | | 0.947 | | | | |
| **Diagnosis with an STI** |  | | |  | | |  | | |
| Follow the doctor’s advice | 0.75 (0.33 – 1.69) | | | | 0.933 | | | | |
| Self-medicate with OTC drugs**^c^** | 1.22 (0.83 – 1.80) | | | | 0.747 | | | | |
| Seek herbal and traditional medicine | 0.96 (0.68 – 1.35) | | | | 0.971 | | | | |
| Ignore the diagnosis if mild | 0.97 (0.64 – 1.48) | | | | 0.971 | | | | |

**^a^ Chi-square test** was utilized to test for association with a 0.05 cut-off point for statistical significance.
**^b^ Benjamin-Hochberg procedure** was utilized to adjust for multiple tested and reduce the false discovery rate to 5% within each family of hypothesis testing (within each individual independent variable).
**^c^** OTC = over the counter.
**^d^** These items showed statistically significant associations before adjusting for multiple testing.
